# Measuring Stress, Socialization, and Smoking Behaviors Among Lesbian, Gay, Bisexual, Transgender, Queer, and Other Sexual and Gender Minority Adolescents (the Puff Break Research Study): Protocol for a Ecological Momentary Assessment Study

**DOI:** 10.2196/71927

**Published:** 2025-07-03

**Authors:** Linda Salgin, Daniel Kellogg, Jonathan Helm, Aaron J Blashill, Mark Myers, Hee-Jin Jun, Andy C Lim, Jerel P Calzo

**Affiliations:** 1 San Diego State University and University of California San Diego Joint Doctoral Program in Public Health San Diego United States; 2 San Diego State University Research Foundation San Diego, CA United States; 3 Department of Psychology College of Sciences San Diego State University San Diego, CA United States; 4 San Diego State University and University of California San Diego Joint Doctoral Program Clinical Psychology San Diego, CA United States; 5 VA San Diego Healthcare System and Department of Psychiatry University of California San Diego San Diego, CA United States; 6 School of Public Health San Diego State University San Diego, CA United States

**Keywords:** telemedicine, mobile app, vaping, cigarette smoking, cannabis, nicotine, smoking, sexual and gender minority adolescents

## Abstract

**Background:**

Adolescent tobacco and nicotine use is a major public health concern, with lesbian, gay, bisexual, transgender, queer, and other sexual and gender minority (LGBTQ+) adolescents showing disproportionately high use compared to their heterosexual and cisgender peers. Research suggests factors such as socialization, stress, mood, and craving exacerbate tobacco and nicotine use. However, there is a dearth of knowledge of how these factors influence tobacco, nicotine, and cannabis use among LGBTQ+ adolescents in general and particularly on a momentary basis.

**Objective:**

This study aims to use ecological momentary assessment (EMA) to assess real-time predictors of tobacco, nicotine, and cannabis product use among LGBTQ+ adolescents.

**Methods:**

The Puff Break protocol was adapted from existing EMA protocols, key informant recommendations, LGBTQ+ adolescent perspectives, and insights from community members. Recruitment occurred through multiple channels, with high recruitment results via social media. Eligible participants were aged 14 to 19 years; self-identified as LGBTQ+; and used tobacco, nicotine, or cannabis products at least once in the past 30 days. The EMA pilot began with a 1.5-hour in-person or remote meeting where participants completed a timeline follow-back assessment for tobacco and nicotine use, salivary cotinine assessment, baseline survey, and EMA protocol training. Then, participants completed a 2-week EMA trial during which they received 1- to 2-minute surveys 5 times a day. Within a week of completing the EMA trial, participants concluded with an exit survey and exit interview.

**Results:**

Funded in July 2022, the Puff Break study conducted EMA data collection between August 2023 and November 2024, recruiting a sample of 50 participants. Analyses evaluating the feasibility and acceptability of the Puff Break EMA protocol will be completed by July 2025. Multilevel modeling techniques to estimate both contemporaneous and lagged associations among stress, socialization, and craving (exposures) and smoking (outcomes—combustible cigarette, smokeless product, e-cigarette, and cannabis use) are expected to be completed by November 2025. Finally, qualitative thematic analyses to identify robust tailoring variables, intervention options, and decision rules to support future just-in-time-adaptive intervention development are expected to be completed by May 2026.

**Conclusions:**

Puff Break is an innovative EMA protocol developed to capture factors influencing tobacco, nicotine, and cannabis use among LGBTQ+ youth. Despite some inherent limitations to the EMA design, the Puff Break protocol has the potential to inform the development of a just-in-time-adaptive intervention to reduce tobacco, nicotine, and cannabis use among LGBTQ+ adolescents.

**International Registered Report Identifier (IRRID):**

DERR1-10.2196/71927

## Introduction

### Background

Adolescent and young adult tobacco and nicotine use continues to be a major public health concern. The 2024 National Youth Tobacco Survey found that 8.1% of all middle and high school youths surveyed reported current use of tobacco products, with 2.8% of students reporting combustible cigarette use, while 5.9% of middle school students and 7.8% of high school students reported current e-cigarette use [1]. While rates of e-cigarette use declined from the previous year, demonstrating the success of public health efforts and policy regulations to prevent or reduce use among youth (eg, warning labels, and drug and tobacco policies to regulate sales and access) [2,3], 38.4% of the students using e-cigarettes reported frequent use (at least 20 out of 30 days) and 26.3% reported daily use [4]. Similar studies, such as Monitoring the Future, also highlight that, in 2024, 9% of 8th graders, 12.7% of 10th graders, and 19.5% of 12th graders reported using any nicotine products [5]. Adolescent and young adult users of e-cigarettes have an elevated likelihood of initiating combustible cigarette use [6,7] and using e-cigarettes in conjunction with other tobacco products (cigars, and smokeless tobacco) [8-10], thus increasing their risk for nicotine dependence.

Early prevention of tobacco use in all forms is paramount. It is particularly imperative to target research and prevention efforts toward subpopulations who are at elevated risk for both smoking and tobacco-related disease and negative consequences, such as lesbian, gay, bisexual, transgender, queer, and other sexual and gender minority (LGBTQ+) adolescents [11,12]. Starting in adolescence, LGBTQ+ populations are at substantially elevated risk for combustible tobacco and e-cigarette use compared to heterosexual and cisgender populations [13,14]. Data from the California Healthy Kids Survey (CHKS) shows that middle and high school LGBTQ+ students report a 1% to 2% higher prevalence of past 30-day current use of combustible cigarettes relative to their heterosexual peers, and high school transgender students report a 6% to 7% higher prevalence of past 30-day current use of combustible cigarettes relative to their cisgender peers [15]. Similarly, middle and high school LGBTQ+ students report a 2% to 5% higher prevalence of past 30-day current use of e-cigarettes relative to their heterosexual peers, and high school transgender students report a 7% higher prevalence of past 30-day current use of e-cigarettes relative to their cisgender peers [15]. Furthermore, an analysis of CHKS data revealed that transgender middle and high school students of color had up to 6.0 greater odds of using e-cigarettes or vape products in the last 30 days relative to their cisgender peers of the same race and ethnicity [16]. Similar studies using nationally representative and longitudinal cohort data also highlight increased prevalence and odds of tobacco and nicotine use among LGBTQ+ adolescents relative to their heterosexual and cisgender peers [17,18]. While the overall prevalence of combustible cigarettes and e-cigarette use is decreasing among LGBTQ+ adolescents, addressing nicotine and tobacco dependence and addiction is still an important health priority [19].

Beyond typical risk factors for smoking in adolescence (eg, mood, craving, risk perceptions and expectancies, exposure to tobacco-related media, and being around peers who smoke) [20-22], sexual orientation and gender disparities in smoking may be further elevated due to daily minority stress and LGBTQ+-specific socialization experiences. Minority stress theory [23-25] posits that LGBTQ+ populations experience excess stress due to exposure to various forms of prejudice, discrimination, and internalized stigma, which results in adverse mental and physical health (eg, anxiety and depression) and maladaptive coping behaviors (eg, smoking and vaping). Elevated rates of smoking have been associated with high degrees of sexuality- and gender-based mistreatment. In their analysis of CHKS data, Coulter et al [26] found that sexual and gender minority middle and high school students reported substantially higher rates of sexuality- and gender-based harassment than their heterosexual and cisgender peers. The combined effect of sexuality- and gender-based harassment led to significantly higher tobacco and e-cigarette use compared to when each type of harassment was evaluated separately [26].

In addition to stress-related factors, LGBTQ+ individuals may be more likely to smoke due to socialization factors, such as perceived norms that promote tobacco use [27,28]. For instance, a review by Simons-Morton and Farhat [27] found that peer group homogeneity (eg, adolescents with friends who smoke are more likely to smoke themselves), peer influence (eg, best friends who smoke), and crowd affiliation (eg, group membership) predict adolescent smoking behaviors. In a similar light, research by Garcia et al [29] found that adolescents were 7 times more likely to have ever used an e-cigarette if their close friends also used e-cigarettes. Furthermore, studies by Hinds et al [28] indicated that sexual minority young adults are more accepting of cigarette-related norms compared to their heterosexual peers [28,29]. These findings allude to the need to explore how peer group norms may influence tobacco and nicotine product use among LGBTQ+ adolescents.

The contributions of minority stress processes and socialization factors on LGBTQ+ youth smoking have been primarily studied via retrospective surveys [28,30], limiting understanding of the real-time impacts of minority stress and socialization effects on smoking behaviors during the developmental period when sexual orientation and gender disparities in smoking emerge. Minority stress experiences [31,32] and exposure to peer norms [27-29] that influence smoking are common daily events. These events shape contemporaneous and future smoking, requiring methods that enable intensive time-series analyses to examine the contributions of these factors to LGBTQ+ adolescent smoking behaviors. Therefore, a deeper understanding of these real-time impacts is crucial for developing effective interventions.

Ecological momentary assessment (EMA) is a moment-to-moment data collection method that can detect real-time relationships between experiences with exposures, such as stress, sociocontextual cues, psychological states, and changes in behavioral outcomes [33], such as tobacco, nicotine, and cannabis product use. EMA research has examined the contributions of minority stress and socialization factors to smoking among LGBTQ+ adult populations [34-36]. For example, Livingston et al [32] found that for every unit increase in stress, there was a 3-fold increase in reports of smoking among LGBTQ+ young adults in the same observation period. EMA research by Nguyen et al [34] also identified sociocontextual and environmental influences (eg, being around other smokers and socializing in bars) as unique risk factors for smoking. However, the study was constrained to only the adult LGBTQ+ population. Identifying age-appropriate sociocontextual and environmental risk (and protective) factors for LGBTQ+ adolescents warrants additional research.

EMA methodologies have been used to measure exposure to tobacco-related media among adolescent students with high rates of retention and participation, demonstrating their potential acceptability and feasibility among this adolescent population [21]. Furthermore, EMA can be adapted for dynamic interventions (eg, just-in-time adaptive interventions [JITAIs]), enabling researchers to both measure substance use behavior and quickly implement interventions in response to triggers for behavior [37]. With limited EMA studies on tobacco and nicotine use among LGBTQ+ adolescents, significant gaps remain in understanding specifically how minority stress and socialization factors jointly contribute to these behaviors at the individual level in real time among LGBTQ+ youth. Delineating such processes could contribute to the development of secondary prevention approaches to reduce current tobacco, nicotine, and cannabis use and promote positive health behaviors (eg, salubrious coping strategies) in response to underlying behavioral determinants.

### This Study

The Puff Break Research Study, further referred to as Puff Break, is a 2-phase EMA pilot designed to characterize the momentary associations among general stress, minority stress, socialization, and other evidence-based predictors of tobacco, nicotine, and cannabis product use (eg, mood, craving) among LGBTQ+ adolescents aged 14 to 19 years. In phase 1, Puff Break aimed to use key informant interviews with subject matter experts and LGBTQ+ adolescent interview data to adapt an existing adult EMA protocol to validly and reliably measure minority stress exposure, smoking socialization, and smoking and vaping behavior among LGBTQ+ adolescents. In phase 2, via the adapted EMA protocol, we will assess the acceptability and feasibility of real-time measurement of minority stress; smoking socialization; and tobacco, nicotine, and cannabis product use behaviors in a sample of 50 LGBTQ+ identified adolescents (aged 14-19) who reported combustible cigarette or e-cigarette use. This paper describes the phase 2 protocol.

## Methods

### Ethical Considerations

All study procedures were reviewed and approved by the San Diego State University Human Research Protection Program’s Institutional Review Board (HS-2022-0101).

Puff Break used Tango gift cards to remunerate participants, as Tango gift cards offer a wide variety of redeemable merchants and branded gift cards. Participants received US $25 for completing the baseline survey and US $25 for completing the exit survey and interview. Remuneration during the EMA trial was commensurate with the completion rate of the assessment, in which participants received an additional US $50 for a <50% survey completion rate, US $100 for ≥50% and ≤80%, or an additional US $125 for >80%. The total remuneration a participant could receive ranged from US $100 to US $175. The remuneration schedule is consistent with previous EMA research of similar duration with adolescent samples [38,39]. Participants who attended a meeting in person were given an additional US $40 for transportation reimbursement. Thus, participants who attended both the onboarding and exit meetings in person received an additional US $80.

### Study Development

Phase 1 of the project included the development of the EMA protocol, adapted from other existing protocols for measuring antecedents and correlates of smoking behaviors among LGBTQ+ young adults [32,34,36] and other existing protocols for measuring antecedents and correlates of smoking behaviors among LGBTQ+ young adults. To ensure the acceptability, feasibility, and appropriateness of phase 2 of the Puff Break protocol, we first established a participatory planning group, consisting of 5 leaders within the local county health department and nonprofit organizations focused on serving LGBTQ+ youth, to consult on their expertise related to the goals of the project. In addition, we conducted semistructured interviews with 7 key informant subject matter experts and 10 LGBTQ+ adolescents, 90% (9/10) of whom reported tobacco, nicotine, and cannabis use. Insights from these interviews led to iterative adaptations (eg, measurement selection, preferences for response formats, momentary sampling response schedule, and flexibility of scheduling due to work or school conflicts), contributing to the development of the phase 2 protocol described in this paper.

**Figure 1:**
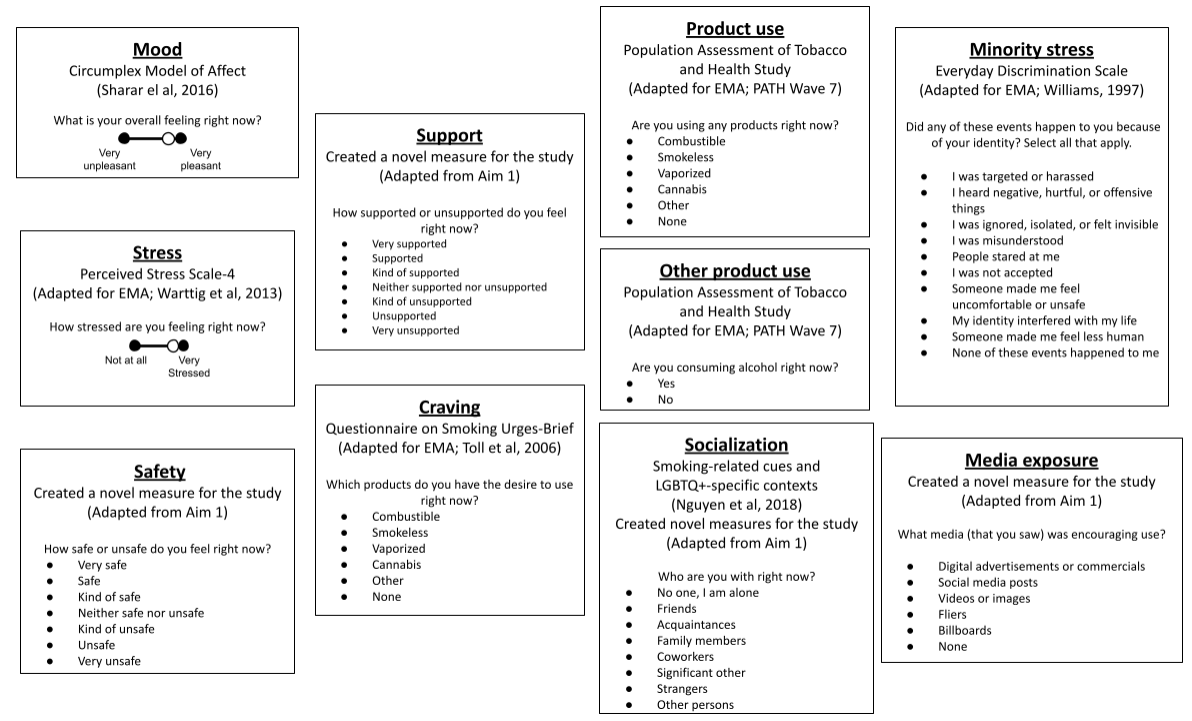
Puff Break ecological momentary assessment (EMA) protocol domains [34,41-45]. Participants reported on the domains of mood, stress, safety, support, and craving in the present moment and the domains of product use, other product use, and socialization in both the present moment and retrospectively. Minority stress and media exposure were reported retrospectively. LGBTQ+: lesbian, gay, bisexual, transgender, queer, and other sexual and gender minority.

The phase 2 protocol was programmed into the mobile EMA (mEMA) software ilumivu [40]. Figure 1 [34,41-45] displays the domains of the final adapted EMA protocol used to assess stress, socialization, and other pertinent factors in relation to tobacco or nicotine product use among LGBTQ+ adolescents. A detailed list of measures included in the EMA protocol is provided in Multimedia Appendix 1 [41,46-56] and Multimedia Appendix 2 [34,41-43,46,55,57-61]. Both in-person and remote protocols to train participants on EMA procedures and complete onboarding and exit assessments were developed to make the study as accessible as possible for participants. Unless otherwise noted, study components were identical for the in-person and remote versions of Puff Break.

### Recruitment Efforts

#### Overview

To pilot the adapted EMA protocol, we recruited 50 LGBTQ+ adolescents with different sexual orientations and gender identities between August 2023 and November 2024. Participants were recruited using multiple methods, including distributing flyers, tabling at local youth and LGBTQ+ community events, presenting to community and academic partners, participant snowball sampling, and social media posts.

#### Flyers

Seven flyers were developed by the Puff Break team to appeal to different social identities of LGBTQ+ adolescents, with a brief description of Puff Break and a QR code to the screener survey. On the basis of previous experiences engaging LGBTQ+ adolescents in research and recommendations from the participatory planning group, flyers were posted in local university settings (eg, the student union, student housing office, and the campus LGBTQ+ center) and local community centers or storefronts (eg, youth LGBTQ+ drop-in centers, libraries, and coffee shops) that served or were accessed by youth.

#### Community Events and Presentations

The Puff Break team conducted 8 presentations to community and academic partners and 3 tabling events where staff shared information regarding the study and shared flyers with potentially eligible participants.

#### Referrals

Enrolled participants provided recommendations on potential peers who might be eligible and interested in joining the study.

#### Social Media

Using the social media platforms Facebook and Instagram (Meta Platforms, Inc), advertisement campaigns were created and configured to target specific age groups and demographics. Advertisement campaigns were reconfigured iteratively to target specific participants, such as updating advertisement artwork to appeal to younger audiences and expanding the campaign’s geographic radius to reach additional communities of color disproportionately impacted by tobacco and nicotine use. As social media platforms, such as Facebook and Instagram, do not allow researchers to target specific demographic groups, especially minors, we also attached relevant keywords to appeal to our target population. Available keywords relevant to LGBTQ+ culture (eg, Pose, Charlie XCX, Love, Simon, and gender) were attached to the campaign. The advertisements consisted of 1 of the 7 flyers, along with a short statement in the textbox of the advertisement’s post that encouraged participation in the study. A link to the screener survey was provided for participants to submit their responses for determining eligibility. Each week, a Puff Break team member manually edited the campaign to run a new advertisement cycle. After comparing views and click rates across weekdays and weekends, the team chose to run advertisements every Thursday through Sunday, and the advertisement costs varied from US $25 to US $75, in total, per advertisement life cycle.

### Screener Survey

The following inclusion criteria were set based on previous EMA studies on youth smoking behavior [22,65]: (1) individuals who reported current tobacco, nicotine, or cannabis product use (ie, any use in the past 30 days); (2) individuals who were aged between 14 to 19 years old; (3) individuals who self-identified as LGBTQ+; and (4) individuals who had daily access to a personal smartphone. The screener survey collected respondents’ contact and demographic information (age, residence, race, ethnicity, sex, gender identity, and sexual orientation). The survey also included 13 questions assessing the past month and daily product use. Participants were first asked if they had used any of the following products within the past 30 days: combustible cigarette, cigar, cigarillo, bidi, hookah, e-cigarette, chewing tobacco, snus, snuff, nicotine lozenges, nicotine patches, nicotine gum, cannabis, and other. If they responded yes to using any of the products, additional follow-up questions were asked, adapted from the Population Assessment of Tobacco and Health study, Wave 7 [41]. However, a more detailed assessment of recent product use was captured for those who enrolled in the study at the onboarding meeting (refer to Timeline Follow Back Assessment section). Finally, participants were asked to choose a potential date for onboarding into the study, provided they met the eligibility criteria. Participants who reported using combustible or vaporized products at least 5 times per day on a typical day were prioritized for enrollment, but this level of use was not an eligibility requirement.

### Pilot Study Components

**Figure 2:**
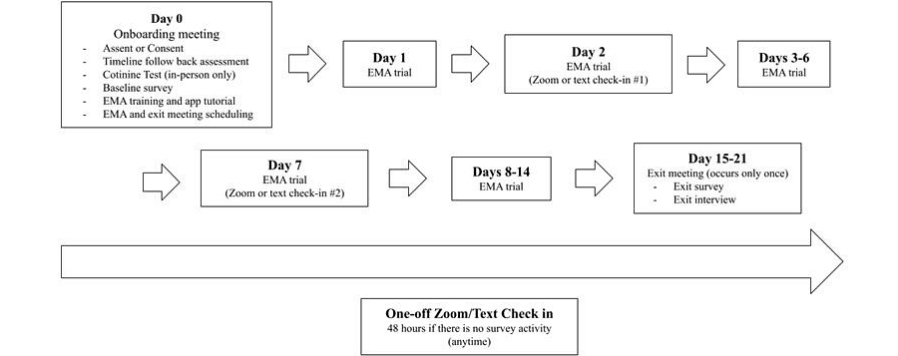
The Puff Break study schedule. The Exit Meeting occurred as soon as Day 15 and as late as Day 21 due to participant scheduling availability. Zoom or text check-ins were scheduled at any time between Day 1 and Day 14, only if participants did not submit any surveys within 48 hours. Check-in communication methods depended on participant preference.

The overall study components included an in-person or remote onboarding meeting, a 2-week EMA trial, and an in-person or remote exit meeting (Figure 2). Except when noted, in-person and remote protocols were identical.

#### Onboarding Meeting

##### Overview

Once participants completed the screening form and were deemed eligible, they were invited to enroll into Puff Break at an in-person or remote onboarding meeting with a study research assistant. Participants were contacted 24 hours before the onboarding meeting to confirm participation. The onboarding meeting consisted of the following: (1) consent or assent, (2) a 2-week timeline follow-back assessment, (3) salivary cotinine test using NicoTest test strips (in-person protocol only), (4) baseline survey, (5) training on the EMA protocol, and (6) tutorial on how to use the EMA app (mEMA powered by ilumivu). Participants were also asked to schedule 2 check-in meetings during their EMA trial as well as their final exit meeting.

##### Identity Verification

Before consent or assent, participants were asked to verify their identity via a state-issued driver’s license, a school-based ID, or a passport. Identity verification was used to ensure the participant matched their screener survey responses, as some online screeners were populated with disingenuous responses or completed by bots. Participants who used a preferred name or had a gender expression different from that on their ID were included in the study. Participants who were unable to verify their identity were offered an opportunity to reschedule the meeting when identification was possible. For remote onboarding, participants were instructed to turn their cameras on and show their IDs via camera to verify their identity. To ensure participant confidentiality and comfort during the Zoom (Zoom Communications, Inc) meeting, participants were encouraged to sit in a private, indoor place where they would not be disturbed; wear headphones; and have their computer facing a wall.

##### Assent and Consent Form

During the assent (for participants aged 14-17 years old) and consent (for participants aged >18 years old) process, the study research assistant reviewed the form with the participant and answered any questions the participant had. For in-person meetings, the participants signed and dated a physical copy of the assent or consent form that was then signed by a Puff Break research assistant as a witness. For remote onboarding, the assent and consent forms were integrated into a Qualtrics (Qualtrics International Inc) survey, which was provided to the participants via the Zoom chat feature. Once the form was reviewed, the participants typed their full name and date in the survey’s respective textboxes, along with the name of the Puff Break research assistant who was with them before submitting the survey. The Puff Break research assistant confirmed receipt of the signed assent or consent form before proceeding with the onboarding meeting. Due to the potential risk of “outing” LGBTQ+ youth and disclosing unknown youth-smoking behaviors to their parents and guardians, a waiver of parental consent was authorized by the San Diego State University Institutional Review Board.

##### Timeline Follow-Back Assessment

The 2-week timeline follow-up assessment assessed the participants’ product use over the past 2 weeks before their onboarding meeting. The measure was adapted from the timeline follow back questions asked in the Population Assessment of Tobacco and Health Wave 7 Youth and Parent questionnaire [41]. The Puff Break research assistant shared a document with pictures of each product (ie, combustible cigarette, cigar, cigarillo, bidi, hookah, e-cigarette, chewing tobacco, snus, snuff, lozenges, nicotine patches, nicotine gums, cannabis, and other) with the participant to reference when answering each question. Product-by-product, the Puff Break research assistant asked if the participant had used the product within the 2-week period. If the participant said “yes” to using a product, they were asked to specify which day or days within the 2-week period they used the product. As the specific day or days were determined on the calendar, the Puff Break research assistant privately filled out the assessment via a Qualtrics matrix. The matrix consisted of the date of onboarding; the various tobacco, nicotine, and cannabis products of interest; and checkbox response options inclusive of the following: has not used, used today, 1 day ago, 2 days ago, 3 days ago, 4 days ago, 5 days ago, 6 days ago, 7 days ago, 8 days ago, 9 days ago, 10 days ago, 11 days ago, 12 days ago, 13 days ago, and 14 days ago. Once the matrix was populated, the Puff Break research assistant verbally confirmed all the participants’ answers before submitting the survey.

##### Salivary Cotinine Test

For biochemical validation of self-reported tobacco and nicotine use, we measured cotinine levels using the NicoTest Extra-Sensitive Nicotine Saliva 30 ng Test Kit [62] that required participants to submit a saliva sample to be pipetted on a test strip. Participants were instructed to drink 8 ounces of water and wait approximately 10 minutes, as indicated in the manufacturer’s instructions, before submitting the saliva sample. Once the results were ready (approximately in 5 min), the Puff Break research assistant privately recorded the test result via a Qualtrics survey. If the first NicoTest result was invalid, a second test was repeated. If the second test was invalid, it was excluded from the study. The NicoTest was excluded for remote participants.

##### Baseline Survey

The baseline survey was administered via Qualtrics, where the participants self-reported their responses on a study-provided iPad (Apple Inc). The baseline survey consisted of several blocks of detailed questions (eg, demographics, past product use [lifetime and 30 days], past cannabis-specific use, minority stress assessment, socialization, product purchasing, marketing, social media exposure, motivations to use products, product dependency, and mood). Details of the baseline measure are shown in Multimedia Appendix 1.

##### EMA Training and EMA App Tutorial

The EMA training and EMA app tutorial were combined into 1 slide deck presented to the participant by a Puff Break research assistant. The EMA training consisted of the origins of EMA, general descriptions of the EMA survey question blocks, the safety and security of the mEMA app, how to report product use, the daily survey schedule, and the schedule of the entire Puff Break protocol. For the tutorial, the Puff Break research assistant instructed the participant on how to download the app, helped them select the optimal settings for their mobile device, and showed them where to access their surveys within the app. Participants were also shown how to update the app and were provided with a test survey to practice answering questions.

##### EMA and Exit Scheduling

Once the participant completed the EMA app tutorial, 2 check-ins with the Puff Break research team were scheduled for the second and seventh days of the EMA trial. Participants were also offered to adjust their survey schedule by an hour per survey if they had known scheduling conflicts (eg, class or work). For example, a participant could delay their 9 AM survey to 10 AM or prompt their 6 PM survey to 5 PM. Even with these adjustments, participants still had a minimum of 2 hours scheduled in between each survey. The Puff Break study staff also used this time to schedule the participants’ final exit meeting, no later than 1 to 2 weeks after their 2-week EMA trial participation ended.

#### EMA Trial and Measures

The 2-week EMA trial consisted of 5 surveys, approximately 1 to 2 minutes in length, delivered to the participant’s mEMA app between 8:30 AM and 9:30 PM, on a 2- or 3-hour cycle, depending on their survey schedule. Using the administrative functions of mEMA’s web-based account manager, the survey delivery time was randomly generated, within 30 minutes before or after the standard delivery times of the 5 surveys (9 AM, 12 PM, 3 PM, 6 PM, and 9 PM). Therefore, a 9 AM survey could be delivered as early as 8:30 AM and as late as 9:30 AM. Participants had 60 minutes to submit their survey upon delivery. Participants were informed of their survey delivery via an initial push notification, with 2 additional push notifications prompted 15 and 30 minutes after their survey was delivered, if the survey was not yet complete. All 5 surveys were also programmed into Qualtrics to be used as a backup in case there were technical difficulties with the mEMA app; however, the Puff Break team never used this backup option during the pilot.

As displayed in Figure 1, the question blocks consisted of the following: (1) current mood; (2) current stress, current safety, and support; (3) products currently used; (4) products currently craved; (5) other substances currently used; and (6) current social environment. Retrospectively, participants were also asked about the following: (1) products used since the last survey, (2) other products used since the last survey, (3) experiences of minority stress, (4) the past social environment, and (5) product-related media they had seen. Each survey’s responses were uploaded to Puff Break’s ilumivu cloud. No survey responses were saved on participants’ mobile devices. EMA measures can be found in Multimedia Appendix 2.

Two check-ins were conducted with each participant on the second and seventh day of the EMA trial via Zoom or text, depending on the participant’s preference. A Puff Break research assistant would ask the participant 2 questions: “How is the study going for you so far?” and “Are there any challenges you are experiencing in the study?” If a participant stated that they experienced a challenge, the Puff Break research assistant probed on how they resolved the challenge or helped the participant resolve it. Finally, the Puff Break research assistant would inform the participants of their current retention rate and encourage them to complete as many surveys as possible with the time remaining in the study. Participants were reminded that they could contact the Puff Break staff during working hours if they needed assistance. If a participant did not submit any surveys within a 48-hour period, a Zoom meeting was scheduled to check in with the participant and ensure they were still able and willing to participate in the study. No participant withdrew or was dismissed from the study prematurely.

#### Exit Meeting

##### Overview

At the end of the 2-week EMA period, participants attended a final exit meeting to conclude their participation in the study. The exit meeting consisted of the following: (1) a second NicoTest, (2) an exit survey, and (3) a semistructured interview. Participants were contacted 24 hours before the exit meeting to confirm participation. Exit meetings were held either in person or remotely via Zoom. If participants attended the meeting in person, the NicoTest was offered, using the same guidelines as the onboarding meeting. If participants attended remotely, the NicoTest was excluded from their meeting. Participants then completed a 10- to 15-minute exit survey as well as a 60-minute semistructured exit interview.

##### Exit Survey

The exit survey used both open- and closed-ended survey questions to gather feedback on participants’ experience with the EMA, including the following: (1) satisfaction with the process, (2) the perceived burden of participation in the study, (3) their preferred reporting methods, and (4) and reflections on administrative support (training and technical support). Seven open-ended questions were adapted from the study team’s adult EMA protocol, which included questions such as “What did you like or dislike about the EMA method?” and “What procedures were difficult to complete, confusing, or required additional support?” Closed and open-ended questions assessed the EMA protocol’s acceptability and feasibility. Acceptability was operationalized as the perception among LGBTQ+ adolescents that the Puff Break protocol was appealing, favorable, or satisfactory. Feasibility was operationalized as the extent to which the Puff Break protocol could be successfully implemented among the target population. Measures were adapted from the 12-item Acceptability of Intervention Measure (AIM), Intervention Appropriateness Measure (IAM), and Feasibility of Intervention Measure (FIM) [63]. Each component of the assessment consists of 4 items specific to each domain, measuring the extent to which the participant believes an evidence-based practice is acceptable, appropriate, and feasible. These measures are intended to be as pragmatic as possible and were adapted to the context of Puff Break. The AIM, IAM, and FIM are measured via a 5-point Likert scale where 1 indicates completely disagree and 5 indicates completely agree. In addition, participants completed the 6-item Mobile Application Rating Scale app-specific subscale, which aims to assess the perceived impact of an app on the user’s knowledge, attitudes, and intentions to change as well as the likelihood of actual change in the target health behavior [64,66]. Questions were adapted to fit the context of the Puff Break. Each of the 6 items is measured using a 5-point Likert scale (1=strongly disagree to 5=strongly agree).

##### Exit Interview

The final activity for Puff Break included a 60-minute semistructured interview, with approximately 15 questions wherein participants reflected on their tobacco and nicotine or cannabis product use during the 2-week EMA trial, provided additional feedback on the Puff Break protocol, and provided feedback on how to best disseminate a future mEMA intervention aimed at monitoring and reducing tobacco and nicotine or cannabis product use among this target demographic. The first 8 questions were specific to the EMA responses submitted by the participants. The Puff Break team generated a series of bar charts depicting trends in tobacco, nicotine, and cannabis product use by day of the week and time of day for the participant being interviewed. Each participant was asked to confirm the different products they reported using during the EMA trial and to describe what happened during the given day or time windows that may have affected their product use. Participants were also asked to think about what would have stopped them from using tobacco and nicotine products during those moments. In addition, participants were asked to reflect on their tobacco, nicotine, and cannabis product use depending on their social environments (eg, where they were or who they were with), stressors experienced, and cravings. The next 7 questions were informed by the Reach, Effectiveness, Adoption, Implementation, and Maintenance framework by Glasgow et al [67] to understand how a future EMA mobile intervention aimed at monitoring and reducing tobacco, nicotine, and cannabis product use could effectively be disseminated to, reached by, and implemented with LGBTQ+ adolescents. To assess *reach*, participants were asked to think about how to increase the likelihood that LGBTQ+ adolescents would download an app such as Puff Break (eg, where the app should be advertised and types of messaging [negative or positive] used to advertise the app). *Effectiveness* and *implementation* were assessed by asking participants to reflect on how an app such as Puff Break could affect their ability to monitor their tobacco, nicotine, and cannabis product use; what would motivate them to download an app such as Puff Break; and what features would be needed in the app to increase its effectiveness (eg, trackers, data visualizations, and motivational messages). For *adoption,* participants were asked if they have ever been screened for or advised to quit tobacco and nicotine products, who they would want to hear about this type of app from (eg, friends, family, and trusted individuals), and when would be an ideal time to learn about an app such as Puff Break once developed. Finally, we assessed *maintenance* by asking participants to think about what would motivate them to continue using the app (eg, what makes an app easy or difficult to use).

### Data Management

#### Quantitative

Responses from participants were downloaded from cloud storage to an encrypted, password-protected server accessible only to the research team and manually reviewed and cleaned. After the data were cleaned, they were merged into the existing database and deidentified. While EMA data had a unique user ID within the mEMA program, they were also merged using their participant IDs. A private cloud storage folder that stored a separate key file for participant names, ID numbers, and consent and assent forms was only accessible to project leadership.

#### Qualitative

Exit meeting audio files (from Zoom or in-person recordings) were transcribed verbatim. After transcription, the study team reviewed transcripts against audio files for accuracy, and the audio files were deleted. Transcripts from participant exit meetings were stored on the Puff Break team’s restricted, password-protected drive. Participants’ and interviewers’ names as well as any identifiable information stated by the participant (school name, city names, others’ names, employer’s name, etc), were redacted from the transcript.

### Data Analysis

#### Demographics

Demographic characteristics of our sample populations will be reported using descriptive statistics.

#### Acceptability and Feasibility

The primary analysis for this pilot will be conducted to determine the acceptability and feasibility of the EMA protocol. To determine acceptability, we will conduct a descriptive analysis of the mean survey scores according to the AIM measure [63]. Qualitative content analysis of open-ended response feedback from the exit survey and thematic analysis from participant interviews will also be conducted to assess protocol acceptability.

To determine feasibility, we will conduct a descriptive analysis of sample characteristics and compliance data (ie, number of participants who meet the recommended threshold of completing 80% of the scheduled assessments) along with mean survey scores according to the FIM measure [63]. We will also conduct a content analysis of the open-response feedback from the exit survey and a qualitative analysis of participant interview testimonials regarding protocol feasibility. In addition, we plan to conduct a descriptive analysis of the protocol’s appropriateness using the IAM [63] along with analyzing mean survey scores from the Mobile Application Rating Scale app-specific [64,66] subscale to understand the protocol’s perceived impact on user’s knowledge, attitudes, and intentions to use a protocol such as Puff Break to monitor their tobacco, nicotine, and cannabis product use.

#### Associations Among Stress, Socialization, and Smoking

The sample size for the pilot was set to power analyses to detect small to moderate associations among measures of stress, socialization, and smoking outcomes in the EMA data. To maximize power for the calculation of reliability (eg, intraclass correlations), validity, and detection of associations between exposures and outcomes, analysis of EMA data will be conducted at the level of participant observations. Each participant is expected to provide up to 5 assessments per day for 2 weeks (ie, if a participant completed all 5 assessments per day for 2 weeks, then 70 participant observations multiplied by 50 participants will be equal to 3500 participant observations). Using previous effect estimates of the association between minority stress exposures from an EMA study of LGBTQ+ young adults [34,35], we determined that 50 EMA participants with 14 measurement occasions (ie, 1 per day; n=700 participant observations) would provide adequate power (80%) to detect at least a 1.2-unit increase in the odds of smoking. We will investigate missing data on exposures, covariates, and outcomes by summarizing the degree of missing on each variable and potential patterns of missing (eg, by participants and across variables) using data visualization techniques in R software (version 4.3.3; R Foundation for Statistical Computing). Finally, we will use appropriate approaches to handling missing data, such as multiple imputation, based on patterns of missing data observed and consider sensitivity analysis to evaluate robustness of findings against different plausible missing mechanisms (eg, missing at random and missing not at random) [68]. Participant observation data will be analyzed using multilevel modeling techniques to account for the correlated structure (ie, repeated measures within individuals). We will estimate both contemporaneous and lagged associations between stress and socialization (exposures) and smoking (outcome—combustible cigarette and e-cigarette use). Models with and without adjustment for baseline measures (stress and socialization and demographics) and potential covariates (mood, craving, and expectancies, level of nicotine dependence, and other forms of tobacco use and couse behaviors [eg, cannabis]) will be conducted.

#### Intervention Development

Qualitative thematic analysis from the exit interviews will be used to identify overarching themes related to states of vulnerability or opportunity (ie, internal or contextual states when someone may be susceptible to engage in smoking behavior or positive health behavior change) and identify robust tailoring variables (ie, individual-level information that can be used to individualize when and how to intervene, eg, sexual orientation and gender identity), intervention options (ie, possible actions or treatments that can be delivered at decision points, eg, playing a game on the phone to avoid smoking) [69], and decision rules (which intervention option to offer, given the tailoring variables, and when) [37,70]. These data will support future JITAI intervention development. Analysis of qualitative data will further guide the exploration of quantitative data to examine specific antecedents and correlates of smoking behavior to support future JITAI intervention development.

## Results

Funded in July 2022, Puff Break conducted EMA data collection between August 2023 and November 2024. During the data collection period, we recruited a total sample of 50 participants. Analyses are currently underway. We anticipate completing analyses evaluating the acceptability and feasibility of the Puff Break EMA protocol by July 2025. Multilevel models to estimate both contemporaneous and lagged associations among stress, socialization, and craving (exposures) and smoking (outcomes—combustible cigarette, smokeless product, e-cigarette, and cannabis use) are expected to be completed by November 2025. Finally, qualitative thematic analyses to identify robust tailoring variables, intervention options, and decision rules to support future JITAI development are expected to be completed by May 2026.

## Discussion

### Anticipated Findings

Puff Break is an EMA protocol developed through an iterative process incorporating existing EMA protocols, key informant insights, and perspectives from LGBTQ+ adolescents and community members. This protocol aimed to measure a range of factors influencing tobacco, nicotine, and cannabis use behaviors among LGBTQ+ youth on a momentary basis. Participants were onboarded into the study in person or remotely and completed the Puff Break protocol using flexible momentary sampling schedules. The brief Puff Break assessments were bolstered by an in-depth baseline assessment, an exit survey, and an exit interview, all of which provide the research team with additional opportunities to contextualize and validate EMA data. The quantitative and qualitative data collected from Puff Break have great potential to inform the development of JITAIs to reduce tobacco, nicotine, and cannabis use among LGBTQ+ youth.

### Limitations

There are some inherent limitations in the design of the Puff Break protocol. First, the small sample size may limit our ability to detect individual-level tailoring variables (eg, sexual orientation or gender-specific patterns in response) that could be meaningful for JITAI development. Second, while EMA assessments can generate numerous observations per participant, capturing time, state, and context factors that contribute to real-time and lagged tobacco, nicotine, and cannabis use, the brevity of EMA, by design, may overlook key exposures or modifying factors of product use. To account for these potential limitations, Puff Break used exit interviews to carefully review snapshots of each participant’s response data that enabled participants to further contextualize factors that influenced their tobacco, nicotine, and cannabis use.

### Conclusions

If Puff Break is deemed acceptable and feasible by LGBTQ+ youth participants and is able to support identification of robust exposures linked to tobacco, nicotine, and cannabis use, this protocol has great potential to serve as the basis for the development of a JITAI to reduce tobacco, nicotine, and cannabis use. With such results, we will seek funding to adapt Puff Break from an observational tool into an ecological momentary intervention. However, if analyses indicate that Puff Break did not measure robust exposures linked to tobacco, nicotine, and cannabis use among LGBTQ+ youth nor generate enough substantive information to inform JITAI development, we will further adapt the Puff Break protocol until sufficient evidence is collected.

## Appendix

Multimedia Appendix 1 Baseline survey measures and adaptations (when applicable) in the Puff Break study.

Multimedia Appendix 2 EMA survey measures and adaptations (when applicable) in the Puff Break study.

## Abbreviations

AIM: Acceptability of Intervention Measure
CHKS: California Healthy Kids Survey
EMA: ecological momentary assessment
FIM: Feasibility of Intervention Measure
IAM: Intervention Appropriateness Measure
JITAI: just-in-time adaptive intervention
LGBTQ+: lesbian, gay, bisexual, transgender, queer, and other sexual and gender minority
mEMA: mobile ecological momentary assessment

## Data Availability

The datasets generated or analyzed during this study are not publicly available due to organizational policies but are available from the corresponding author on reasonable request.

## References

[1] Results from the Annual National Youth Tobacco Survey. US Food and Drug Administration.

[2] Mylocopos G, Wennberg E, Reiter A, Hébert-Losier A, Filion KB, Windle SB, Gore G, O'Loughlin JL, Grad R, Eisenberg MJ (2024). Interventions for preventing e-cigarette use among children and youth: a systematic review. Am J Prev Med.

[3] Reiter A, Hébert-Losier A, Mylocopos G, Filion KB, Windle SB, O'Loughlin JL, Grad R, Eisenberg MJ (2024). Regulatory strategies for preventing and reducing nicotine vaping among youth: a systematic review. Am J Prev Med.

[4] Park-Lee E, Jamal A, Cowan H, Sawdey MD, Cooper MR, Birdsey J, West A, Cullen KA (2024). Notes from the field: e-cigarette and nicotine pouch use among middle and high school students - United States, 2024. MMWR Morb Mortal Wkly Rep.

[5] Miech RA, Johnston LD, Patrick ME, O’malley PM, Bachman JG (2024). Monitoring the future: national survey results on drug use, 1975-2023: overview and detailed for secondary students. The National Institute on Drug Abuse.

[6] Soneji S, Barrington-Trimis JL, Wills TA, Leventhal AM, Unger JB, Gibson LA, Yang J, Primack BA, Andrews JA, Miech RA, Spindle TR, Dick DM, Eissenberg T, Hornik RC, Dang R, Sargent JD (2017). Association between initial use of e-cigarettes and subsequent cigarette smoking among adolescents and young adults: a systematic review and meta-analysis. JAMA Pediatr.

[7] Barrington-Trimis JL, Yang Z, Schiff S, Unger J, Cruz TB, Urman R, Cho J, Samet JM, Leventhal AM, Berhane K, McConnell R (2020). E-cigarette product characteristics and subsequent frequency of cigarette smoking. Pediatrics.

[8] Soneji S, Sargent J, Tanski S (2016). Multiple tobacco product use among US adolescents and young adults. Tob Control.

[9] Ross JC, Suerken CK, King JL, Wiseman KD, Noar SM, Wagoner KG, Sutfin EL (2018). Adolescents' first tobacco product: results from a nationally representative survey. Tob Regul Sci.

[10] Kowitt SD, Goldstein AO, Sutfin EL, Osman A, Meernik C, Heck C, Ranney LM (2019). Adolescents' first tobacco products: associations with current multiple tobacco product use. PLoS One.

[11] Apelberg BJ, Corey CG, Hoffman AC, Schroeder MJ, Husten CG, Caraballo RS, Backinger CL (2014). Symptoms of tobacco dependence among middle and high school tobacco users: results from the 2012 National Youth Tobacco Survey. Am J Prev Med.

[12] Rubinstein ML, Rait MA, Sen S, Shiffman S (2014). Characteristics of adolescent intermittent and daily smokers. Addict Behav.

[13] Goldbach JT, Mereish EH, Burgess C (2017). Sexual orientation disparities in the use of emerging drugs. Subst Use Misuse.

[14] Corliss HL, Wadler BM, Jun HJ, Rosario M, Wypij D, Frazier AL, Austin SB (2013). Sexual-orientation disparities in cigarette smoking in a longitudinal cohort study of adolescents. Nicotine Tob Res.

[15] System - public dashboards. The California School Climate, Health, and Learning Survey (CalSCHLS).

[16] Felner JK, Andrzejewski J, Strong D, Kieu T, Ravindran M, Corliss HL (2022). Vaping disparities at the intersection of gender identity and race/ethnicity in a population-based sample of adolescents. Nicotine Tob Res.

[17] Azagba S, Ebling T, de Silva GS (2025). E-cigarette, tobacco, and cannabis vaping among diverse sexual and gender identities in U.S. high school students. Public Health.

[18] Harlow AF, Liu F, Young LE, Coreas SI, Rahman T, Unger JB, Leventhal AM, Barrington-Trimis JL, Krueger EA (2024). Sexual and gender identity disparities in nicotine and tobacco use susceptibility and prevalence: disaggregating emerging identities among adolescents from California, USA. Nicotine Tob Res.

[19] Jenssen BP, Walley SC, Boykan R, Little Caldwell AL, Camenga D, Section on Nicotine and Tobacco Prevention and Treatment, Committee on Substance Use and Prevention (2023). Protecting children and adolescents from tobacco and nicotine. Pediatrics.

[20] Dvorak RD, Waters AJ, MacIntyre JM, Gwaltney CJ (2018). Affect, craving, and cognition: an EMA study of ad libitum adolescent smoking. Psychol Addict Behav.

[21] Scharf DM, Martino SC, Setodji CM, Staplefoote BL, Shadel WG (2013). Middle and high school students' exposure to alcohol- and smoking-related media: a pilot study using ecological momentary assessment. Psychol Addict Behav.

[22] Colvin PJ, Mermelstein RJ (2010). Adolescents' smoking outcome expectancies and acute emotional responses following smoking. Nicotine Tob Res.

[23] Hatzenbuehler ML (2009). How does sexual minority stigma "get under the skin"? A psychological mediation framework. Psychol Bull.

[24] Testa RJ, Habarth J, Peta J, Balsam K, Bockting W (2015). Development of the gender minority stress and resilience measure. Psychol Sex Orientat Gend Divers.

[25] Rich AJ, Salway T, Scheim A, Poteat T (2020). Sexual minority stress theory: remembering and honoring the work of Virginia brooks. LGBT Health.

[26] Coulter RW, Bersamin M, Russell ST, Mair C (2018). The effects of gender- and sexuality-based harassment on lesbian, gay, bisexual, and transgender substance use disparities. J Adolesc Health.

[27] Simons-Morton BG, Farhat T (2010). Recent findings on peer group influences on adolescent smoking. J Prim Prev.

[28] Hinds JT, Loukas A, Perry CL (2019). Explaining sexual minority young adult cigarette smoking disparities. Psychol Addict Behav.

[29] Garcia LC, Vogel EA, Prochaska JJ (2021). Tobacco product use and susceptibility to use among sexual minority and heterosexual adolescents. Prev Med.

[30] Davies M, Lewis NM, Moon G (2019). Differential pathways into smoking among sexual orientation and social class groups in England: a structural equation model. Drug Alcohol Depend.

[31] Balsam KF, Beadnell B, Molina Y (2013). The daily heterosexist experiences questionnaire: measuring minority stress among lesbian, gay, bisexual, and transgender adults. Meas Eval Couns Dev.

[32] Livingston NA, Flentje A, Heck NC, Szalda-Petree A, Cochran BN (2017). Ecological momentary assessment of daily discrimination experiences and nicotine, alcohol, and drug use among sexual and gender minority individuals. J Consult Clin Psychol.

[33] Shiffman S (2014). Conceptualizing analyses of ecological momentary assessment data. Nicotine Tob Res.

[34] Nguyen N, McQuoid J, Ramo D, Holmes LM, Ling PM, Thrul J (2018). Real-time predictors of smoking among sexual minority and heterosexual young adults: an ecological momentary assessment study. Drug Alcohol Depend.

[35] Livingston NA, Flentje A, Brennan J, Mereish EH, Reed O, Cochran BN (2020). Real-time associations between discrimination and anxious and depressed mood among sexual and gender minorities: the moderating effects of lifetime victimization and identity concealment. Psychol Sex Orientat Gend Divers.

[36] McQuoid J, Thrul J, Ozer E, Ramo D, Ling PM (2019). Tobacco use in the sexual borderlands: the smoking contexts and practices of bisexual young adults. Health Place.

[37] Coughlin LN, Nahum-Shani I, Philyaw-Kotov ML, Bonar EE, Rabbi M, Klasnja P, Murphy S, Walton MA (2021). Developing an adaptive mobile intervention to address risky substance use among adolescents and emerging adults: usability study. JMIR Mhealth Uhealth.

[38] Shrier LA, Feldman HA, Black SK, Walls C, Kendall AD, Lops C, Beardslee WR (2012). Momentary affective states surrounding sexual intercourse in depressed adolescents and young adults. Arch Sex Behav.

[39] Shrier LA, Burke PJ, Kells M, Scherer EA, Sarda V, Jonestrask C, Xuan Z, Harris SK (2018). Pilot randomized trial of MOMENT, a motivational counseling-plus-ecological momentary intervention to reduce marijuana use in youth. Mhealth.

[40] mEMA App. ilumivu.

[41] Population Assessment of Tobacco and Health (PATH) study [United States] public-use files (ICPSR 36498). National Addiction & HIV Data Archive Program.

[42] Sharar SR, Alamdari A, Hoffer C, Hoffman HG, Jensen MP, Patterson DR (2016). Circumplex model of affect: a measure of pleasure and arousal during virtual reality distraction analgesia. Games Health J.

[43] Williams DR, Yu Y, Jackson JS, Anderson NB (1997). Racial differences in physical and mental health: socio-economic status, stress and discrimination. J Health Psychol.

[44] Warttig SL, Forshaw MJ, South J, White AK (2013). New, normative, English-sample data for the Short Form Perceived Stress Scale (PSS-4). J Health Psychol.

[45] Toll BA, Katulak NA, McKee SA (2006). Investigating the factor structure of the Questionnaire on Smoking Urges-Brief (QSU-Brief). Addict Behav.

[46] About historical NYTS data and documentation. Centers for Disease Control and Prevention (CDC).

[47] Schrager SM, Goldbach JT, Mamey MR (2018). Development of the sexual minority adolescent stress inventory. Front Psychol.

[48] Scheim AI, Bauer GR (2019). The intersectional discrimination index: development and validation of measures of self-reported enacted and anticipated discrimination for intercategorical analysis. Soc Sci Med.

[49] The tobacco use supplement to the current population survey. US Dept of Commerce, Census Bureau.

[50] Johnson JL, Ratner PA, Tucker RS, Bottorff JL, Zumbo B, Prkachin KM, Shoveller J (2005). Development of a multidimensional measure of tobacco dependence in adolescence. Addict Behav.

[51] Khattab AM, AbdelFattah EB, Abozahra A (2017). Study of smoking habit among soldiers in Cairo Security Forces Hospital. Egypt J Chest Dis Tuberc.

[52] Bold KW, Sussman S, O'Malley SS, Grana R, Foulds J, Fishbein H, Krishnan-Sarin S (2018). Measuring E-cigarette dependence: initial guidance. Addict Behav.

[53] Tucker JS, Shadel WG, Edelen MO, Stucky BD, Kuhfeld M, Hansen M, Cai L (2014). Development of the PROMIS Social Motivations for Smoking item banks. Nicotine Tob Res.

[54] DiFranza JR, Savageau JA, Fletcher K, Ockene JK, Rigotti NA, McNeill AD, Coleman M, Wood C (2002). Measuring the loss of autonomy over nicotine use in adolescents: the DANDY (Development and Assessment of Nicotine Dependence in Youths) study. Arch Pediatr Adolesc Med.

[55] Cox LS, Tiffany ST, Christen AG (2001). Evaluation of the brief questionnaire of smoking urges (QSU-brief) in laboratory and clinical settings. Nicotine Tob Res.

[56] Thompson Er (2007). Development and validation of an internationally reliable short-form of the Positive and Negative Affect Schedule (PANAS). J Cross Cult Psychol.

[57] Posner J, Russell JA, Peterson BS (2005). The circumplex model of affect: an integrative approach to affective neuroscience, cognitive development, and psychopathology. Dev Psychopathol.

[58] Do B, Mason TB, Yi L, Yang CH, Dunton GF (2021). Momentary associations between stress and physical activity among children using ecological momentary assessment. Psychol Sport Exerc.

[59] Ebesutani C, Regan J, Smith A, Reise S, Higa-McMillan C, Chorpita BF (2012). The 10-item positive and negative affect schedule for children, child and parent shortened versions: application of item response theory for more efficient assessment. J Psychopathol Behav Assess.

[60] Osika W, Friberg P, Wahrborg P (2007). A new short self-rating questionnaire to assess stress in children. Int J Behav Med.

[61] Cohen S, Kamarck T, Mermelstein R (1983). A global measure of perceived stress. J Health Soc Behav.

[62] Nicotine home drug test kits. NicoTest.

[63] Weiner BJ, Lewis CC, Stanick C, Powell BJ, Dorsey CN, Clary AS, Boynton MH, Halko H (2017). Psychometric assessment of three newly developed implementation outcome measures. Implement Sci.

[64] Terhorst Y, Philippi P, Sander LB, Schultchen D, Paganini S, Bardus M, Santo K, Knitza J, Machado GC, Schoeppe S, Bauereiß N, Portenhauser A, Domhardt M, Walter B, Krusche M, Baumeister H, Messner E (2020). Validation of the Mobile Application Rating Scale (MARS). PLoS One.

[65] Gwaltney C, Bartolomei R, Colby S, Kahler C (2008). Ecological momentary assessment of adolescent smoking cessation: a feasibility study. Nicotine Tob Res.

[66] Stoyanov SR, Hides L, Kavanagh DJ, Zelenko O, Tjondronegoro D, Mani M (2015). Mobile app rating scale: a new tool for assessing the quality of health mobile apps. JMIR Mhealth Uhealth.

[67] Glasgow RE, Harden SM, Gaglio B, Rabin B, Smith ML, Porter GC, Ory MG, Estabrooks PA (2019). RE-AIM planning and evaluation framework: adapting to new science and practice with a 20-year review. Front Public Health.

[68] Ji L, Li Y, Potter LN, Lam CY, Nahum-Shani I, Wetter DW, Chow S (2023). Multiple imputation of missing data in multilevel ecological momentary assessments: an example using smoking cessation study data. Front Digit Health.

[69] Baskerville NB, Dash D, Wong K, Shuh A, Abramowicz A (2016). Perceptions toward a smoking cessation app targeting LGBTQ+ youth and young adults: a qualitative framework analysis of focus groups. JMIR Public Health Surveill.

[70] Nahum-Shani I, Smith SN, Spring BJ, Collins LM, Witkiewitz K, Tewari A, Murphy SA (2018). Just-in-time adaptive interventions (JITAIs) in mobile health: key components and design principles for ongoing health behavior support. Ann Behav Med.

