# Supplementary material for: Measuring Stress, Socialization, and Smoking Behaviors Among Lesbian, Gay, Bisexual, Transgender, Queer, and Other Sexual and Gender Minority Adolescents (the Puff Break Research Study): Protocol for a Ecological Momentary Assessment Study
Source: JMIR Res Protoc. 2025 Jul 3;14:e71927. doi: 10.2196/71927 (PMC12271964; doi:10.2196/71927)
Supplement: Multimedia Appendix 1 [file resprot_v14i1e71927_app1.docx]

**Appendix 1: Baseline Survey Measures and Adaptations (When Applicable) in the Puff Break Study**

| **Domain** | **Measures** | **Question/Prompt/Construct** | **Response Options/Items** | **Source** | **Adaptations (when applicable)** |
| --- | --- | --- | --- | --- | --- |
|  |  |  |  |  |  |
| Demographics | Age | 1. What is your age? | - 14 years old - 15 years old - 16 years old - 17 years old - 18 years old - 19 years old | N/A; original measure | |
| Demographics | Ethnicity | 2. Are you of Hispanic, Latin, or Spanish ethnicity? Please select all that apply. | - Yes, Hispanic - Yes, Latin - Yes, Spanish - Yes, Mexican or Mexican-American - Yes, Chicano - Yes, Puerto Rican - Yes, Cuban - No, - Another ethnicity (please specify) | N/A; original measure | |
| Demographics | Race | 3. Which of the following categories describes your race? Please select all that apply. | - White - Black or African American - African - Native American or Alaskan Native - Asian - Southeast Asian - Middle Eastern - Jewish - Pacific Islander - Another race (please specify) - I don’t know - I prefer not to answer | N/A; original measure | |
| Demographics | Sex assigned at birth | 4. What was your assigned sex at birth? | - Female - Male - Intersex - Another sex at birth (please specify) | N/A; original measure | |
| Demographics | Gender identity | 5. Understanding that gender can be complex, please mark the gender identity that best describes yours. Please select all that apply. | - Woman - Man - Genderqueer - Nonbinary - Gender fluid - Agender - Another gender identity (please specify) | N/A; original measure | |
| Demographics | Transgender identity | 6. Understanding that gender can be complex, do you identify as transgender (for example, transmasculine, or transfeminine)? | - Yes, transmasculine - Yes, transfeminine - Yes, but neither transmasculine nor transfeminine - No - Another gender identity (please specify) | N/A; original measure | |
| Demographics | Sexual orientation | 7. Understanding that sexual orientation can be complex, which of the following best represents your sexual orientation? Please select all that apply. | - Lesbian - Gay - Bisexual - Queer - Pansexual - Asexual - Questioning - Fluid - Homoflexible - Heteroflexible - Straight (heterosexual) - Another sexual orientation (please specify) | N/A; original measure | |
| Demographics | Straight | 8. *[If* ***straight (heterosexual****) selected for 7]*  Are you exclusively straight? | - Yes - No | N/A; original measure | |
| Demographics | Sexual attraction | 9. Understanding that sexual attraction can be complex, which best describes your feelings of sexual attraction(s)? Please select all that apply. | - Only attracted to women - Mostly attracted to women - Equally attracted to men and women - Only attracted to men - Mostly attracted to men - Only attracted to non-binary individuals - Mostly attracted to non-binary individuals - Equally attracted to men and non-binary individuals - Equally attracted to women and non-binary individuals - Equally attracted to men, women, and non-binary individuals - Other (please specify) | N/A; original measure | |
| Demographics | Relationship status | 10. What is your relationship status? | - Single - In a relationship - Engaged - Another relationship status (please specify) | N/A; original measure | |
| Demographics | Household income | 11. How many family members contribute to your household income? | - 0 - 1 - 2 - 3 - 4 | N/A; original measure | |
| Demographics | Employment status | *12. [if* ***1-4 selected*** *for 11]*  What is the employment status of your first family member? | - Employed - Unemployed - Student - Military - Other (please specify) | N/A; original measure | |
| Demographics | Employment status | 13. *[if* ***2-4 selected*** *for 11]*  What is the employment status of your second family member? | - Employed - Unemployed - Student - Military - Other (please specify) | N/A; original measure | |
| Demographics | Employment status | 14. *[if* ***3-4 selected*** *for 11]*  What is the employment status of your third family member? | - Employed - Unemployed - Student - Military - Other (please specify) | N/A; original measure | |
| Demographics | Employment status | 15. *[if* ***4 selected*** *for 11]*  What is the employment status of your fourth family member? | - Employed - Unemployed - Student - Military - Other (please specify) | N/A; original measure | |
|  |  |  |  |  |  |
| Tobacco, Nicotine, and Cannabis Product Use | Lifetime product use | 18. You mentioned that you started using [LIST OF ALL PRODUCTS INITIATED/USED AT EARLIEST AGE]  [in the past 12 months/at age {EARLIEST AGE}]. Which type of tobacco did you try first? | - Cigarettes - E-cigarettes or other electronic nicotine products - Traditional cigars - Cigarillos - Filtered cigars - Shisha or hookah tobacco - Snus - Smokeless tobacco (such as moist snuff, dip, spit, or chew) - IQOS - Other (specify) - Don’t know - Refused | Population Assessment of Tobacco and Health (PATH) Study, Wave 7, Youth / Parent Questionnaire Data [1] | Asked about lifetime use (at least once) instead of the past 12 months/at age and response options were condensed; lozenges, gums, and patches were added:   - Combustible cigarettes - E-cigarettes - Cannabis (marijuana, pot, weed) - Cigars, cigarillos, and bidis - Hookah - Chewing tobacco, snus, snuff - Lozenges, gums, and patches - Other (please specify) - I don’t know - I prefer not to answer |
| Tobacco, Nicotine, and Cannabis Product Use | Age of first cigarette use | 17. How old were you when you first tried cigarette smoking, even one or two puffs? | - __ years old | Population Assessment of Tobacco and Health (PATH) Study, Wave 7, Youth / Parent Questionnaire [1] | Asked about each selected product response option for Q16, separately. |
| Tobacco, Nicotine, and Cannabis Product Use | Past 30-day product use | 18. You mentioned that you started using [LIST OF ALL PRODUCTS INITIATED/USED AT EARLIEST AGE]  [in the past 12 months/at age {EARLIEST AGE}]. Which type of tobacco did you try first? | - Cigarettes - E-cigarettes or other electronic nicotine products - Traditional cigars - Cigarillos - Filtered cigars - Shisha or hookah tobacco - Snus - Smokeless tobacco (such as moist snuff, dip, spit, or chew) - IQOS - Other (specify) - Don’t know - Refused | Population Assessment of Tobacco and Health (PATH) Study, Wave 7, Youth / Parent Questionnaire [1] | Asked about 30-day use (at least once) instead of the past 12 months/ at age and response options were condensed; lozenges, gums, and patches were added:   - Combustible cigarettes - E-cigarettes - Cannabis (marijuana, pot, weed) - Cigars, cigarillos, and bidis - Hookah - Chewing tobacco, snus, snuff - Lozenges, gums, and patches - Other (please specify) - I don’t know - I prefer not to answer |
| Tobacco, Nicotine, and Cannabis Product Use | When products were last used | 19. When was the last time you smoked a cigarette, even one or two puffs? | - Earlier today - Not today but sometime in the past 7 days - Not in the past 7 days but sometime in the past 30 days - Not in the past 30 days but sometime in the past 6 months - Not in the past 6 months but sometime in the past year - 1 to 4 years ago - 5 or more years ago - I don’t know - I prefer not to answer | Population Assessment of Tobacco and Health (PATH) Study, Wave 7, Youth / Parent Questionnaire [1] | Asked about each selected product response option for Q18, separately, around a 30-day timeframe:   - Today - Within the past week - Within the past month - Over a month ago - I don’t know - I prefer not to answer |
| Tobacco, Nicotine, and Cannabis Product Use | Amount of cigarettes per day | 20. In the past 30 days, on the days you smoked, how many cigarettes did you smoke per day? | - Less than 1 cigarette per day - 1 cigarette per day - 2 to 5 cigarettes per day - 6 to 10 cigarettes per day - 11 to 20 cigarettes per day - More than 20 cigarettes per day - I don’t know - I prefer not to answer | Population Assessment of Tobacco and Health (PATH) Study, Wave 7, Youth / Parent Questionnaire [1] | Changed response options:   - 0-4 cigarettes - 5-9 cigarettes - 10-14 cigarettes - 15-19 cigarettes - 20-29 cigarettes - 30+ cigarettes - Other (please specify) - I don’t know - I prefer not to answer |
| Tobacco, Nicotine, and Cannabis Product Use | Weekday cigarette amount | 21. How many combustible cigarettes do you smoke on typical weekdays? | - (Free response) | N/A; original measure | |
| Tobacco, Nicotine, and Cannabis Product Use | Weekend cigarette amount | 22. How many combustible cigarettes do you smoke on typical weekends? | - (Free response) | N/A; original measure | |
| Tobacco, Nicotine, and Cannabis Product Use | Use after waking (cigarettes) | 23. How soon after you wake up do you want to use tobacco? | - Within 5 minutes - From 6 to 30 minutes - From more than 30 minutes to 1 hour - After more than 1 hour but less than 24 hours - I rarely want to use tobacco - Don’t know - Refused | Population Assessment of Tobacco and Health (PATH) Study, Wave 7, Youth / Parent Questionnaire [1] | Asked about combustible cigarettes specifically and the response options were changed:   - 0-5 minutes - 6-15 minutes - 16-30 minutes - 31-60 minutes - 1-2 hours - Over 2 hours - Other (please specify) - I don’t know - I prefer not to answer |
| Tobacco, Nicotine, and Cannabis Product Use | Obtaining cigarettes | 24. In the past 30 days, how did you usually get your own cigarettes? | - I gave someone else money to buy cigarettes for me - I asked someone to give me a cigarette - Someone offered me a cigarette - I took cigarettes from a store or another person - I got cigarettes some other way (specify) - Don’t know - Refused | Population Assessment of Tobacco and Health (PATH) Study, Wave 7, Youth / Parent Questionnaire [1] | Added response options:   - I bought them myself - I got them from a friend - I got them from a family member |
| Tobacco, Nicotine, and Cannabis Product Use | Amount of e-cigarette use | 25. In the past 30 days, on the days you smoked, how many cigarettes did you smoke per day? | - Less than 1 cigarette per day - 1 cigarette per day - 2 to 5 cigarettes per day - 6 to 10 cigarettes per day - 11 to 20 cigarettes per day - More than 20 cigarettes per day - I don’t know - I prefer not to answer | Population Assessment of Tobacco and Health (PATH) Study, Wave 7, Youth / Parent Questionnaire [1] | Asked how many times a day e-cigarettes are used; responses mirrored adapted responses for Q20:   - 0-4 times a day - 5-9 times a day - 10-14 times a day - 15-19 times a day - 20-29 times a day - 30+ times a day - Other (please specify) - I don’t know - I prefer not to answer |
| Tobacco, Nicotine, and Cannabis Product Use | Weekday e-cigarette amount | 26. On average, how many times per day do you use an e-cigarette on typical weekdays? | - (Free response) | N/A; original measure | |
| Tobacco, Nicotine, and Cannabis Product Use | Weekend e-cigarette amount | 27. On average, how many times per day do you use an e-cigarette on typical weekends? | - (Free response) | N/A; original measure | |
| Tobacco, Nicotine, and Cannabis Product Use | Amount of e-cigarette puffs | 28. How many puffs from an electronic nicotine product [have/did] you [taken/take] | - (Free response) | Population Assessment of Tobacco and Health (PATH) Study, Wave 7, Youth / Parent Questionnaire [1] | Changed to ask about the average puff taken whenever they use an e-cigarette   - (Free response) |
| Tobacco, Nicotine, and Cannabis Product Use | Use after waking (e-cigarettes) | 29. How soon after you wake up do you want to use tobacco? | - Within 5 minutes - From 6 to 30 minutes - From more than 30 minutes to 1 hour - After more than 1 hour but less than 24 hours - I rarely want to use tobacco - Don’t know - Refused | Population Assessment of Tobacco and Health (PATH) Study, Wave 7, Youth / Parent Questionnaire [1] | Asked about e-cigarettes specifically and the response options were changed:   - 0-5 minutes - 6-15 minutes - 16-30 minutes - 31-60 minutes - 1-2 hours - Over 2 hours - Other (please specify) - I don’t know - I prefer not to answer |
| Tobacco, Nicotine, and Cannabis Product Use | Obtaining e-cigarettes | 30. In the past 30 days, how did you usually get your own cigarettes? | - I gave someone else money to buy cigarettes for me - I asked someone to give me a cigarette - Someone offered me a cigarette - I took cigarettes from a store or another person - I got cigarettes some other way (specify) - Don’t know - Refused | Population Assessment of Tobacco and Health (PATH) Study, Wave 7, Youth / Parent Questionnaire [1] | Asked about e-cigarettes instead; added response options:   - I bought them myself - I got them from a friend - I got them from a family member |
| Tobacco, Nicotine, and Cannabis Product Use | E-cigarette product purchasing | 31. During the past 30 days, which of the following e-cigarette product(s) did you get or buy from another person? (Select one or more) | - A new e-cigarette device (including disposable devices) - A pod, cartridge, or e-liquid refill - A hit or a drag from another person’s e-cigarette device - Something else (specify) | National Youth Tobacco Survey (NYTS) 2022 Questionnaire [2] | No adaptations |
| Tobacco, Nicotine, and Cannabis Product Use | E-cigarette flavor | 32. In the past 30 days, were any of the e-cigarettes you used flavored? Please select all that apply.  32. What flavors were the e-cigarettes that you have used in the past 30 days? (Select one or more) | - Yes - No - Don’t Know - Menthol - Mint - Clove or spice - Fruit - Chocolate - Alcoholic drinks (such as wine, margarita, or other cocktails) - Candy, desserts, or other sweets - Some other flavor not listed here (Specify:) | National Youth Tobacco Survey (NYTS) 2022 Questionnaire [2] | Combined into one question and shortened response options   - Yes, fruit, candy, mint/menthol - Yes, tobacco, - No - Other (please specify) |
| Tobacco, Nicotine, and Cannabis Product Use | Easier product to smoke | 33. Do you think cigarettes flavored like menthol or mint are easier to smoke, about the same, or harder to  smoke than regular cigarettes? | - Easier to smoke - About the same - Harder to smoke - I don’t know - Don’t know - Refused | Population Assessment of Tobacco and Health (PATH) Study, Wave 7, Youth / Parent Questionnaire [1] | Reworded the question to ask about other flavors than menthol and mint and reworded the responses:  Do you think flavored e-cigarettes (other than menthol or mint) are easier to use, about the same, or harder to use than tobacco-flavored or unflavored e-cigarettes?   - Easier to use than tobacco-flavored or unflavored e-cigarettes - About the same - Harder to use than tobacco-flavored or unflavored e-cigarettes - Other (please specify) - I don’t know - I prefer not to answer |
| Tobacco, Nicotine, and Cannabis Product Use | Preferred e-cigarette product | 34. Do you currently have a preferred type(s) of e-cigarette that you use? Please select all that apply. | - Yes, pod-based - Yes, disposable - Yes, refillable - Yes, modifiable - No - Other (please specify) - I don’t know - I prefer not to answer | N/A; original measure | |
| Tobacco, Nicotine, and Cannabis Product Use | Marijuana and e-cigarette co-use (lifetime) | 35. Have you ever used marijuana in an e-cigarette, vape pen, or electronic nicotine product, even one or two times? | - Yes - No - Don’t know - Refused | Population Assessment of Tobacco and Health (PATH) Study, Wave 7, Youth / Parent Questionnaire [1] | Said “cannabis” instead of “marijuana” |
| Tobacco, Nicotine, and Cannabis Product Use | Marijuana and e-cigarette co-use (30 days) | 36. In the past 12 months, have you used marijuana in an e-cigarette, vape pen, or electronic nicotine  product, even one or two times? | - Yes - No - Don’t know - Refused | Population Assessment of Tobacco and Health (PATH) Study, Wave 7, Youth / Parent Questionnaire [1] | Asked about the past 30 days instead of the past 12 months |
|  |  |  |  |  |  |
| Cannabis Product Use | Amount of cannabis use | 37. On how many of the past 30 days did you use marijuana in an electronic nicotine product? | - ___ days - Don’t know - Refused | Population Assessment of Tobacco and Health (PATH) Study, Wave 7, Youth / Parent Questionnaire [1] | Changed to ask how many times cannabis specifically is used, regardless of method. Response options match Q20:   - 0-4 times a day - 5-9 times a day - 10-14 times a day - 15-19 times a day - 20-29 times a day - 30+ times a day - Other (please specify) - I don’t know - I prefer not to answer |
| Cannabis Product Use | Weekday cannabis amount | 38. On a typical weekdays, how many times do you use cannabis? | - (Free response) | N/A; original measure | |
| Cannabis Product Use | Weekend cannabis amount | 39. On a typical weekends, how many times do you use cannabis? | - (Free response) | N/A; original measure | |
| Cannabis Product Use | Cannabis method | 40. How do you use cannabis? Please select all that apply. | - Smoke - Vaporize - Edibles - Tincture - Dabs - Plants - Other (please specify) - I don’t know - I prefer not to answer | N/A; original measure | |
| Cannabis Product Use | Medical purposes | 41. Do you have a physician's or psychiatrist's recommendation to use cannabis for medical purposes? | - Yes, - Yes, but I use cannabis for both medical and non-medical purposes - No - Other (please specify) - I don’t know - I prefer not to answer | N/A; original measure | |
|  |  |  |  |  |  |
| Minority Stress | Sexual orientation-based stress | 42. Please read each statement and answer “Yes” if it has ever happened to you in the past, or “No”  if it hasn’t. If you said “Yes” to a statement, please also answer the follow-up question about whether it is  currently happening. For the follow-up questions, you should answer “Yes” if it happened to you within the  past 30 days, or “No” if it happened to you more than 30 days ago.  You should select the one option that best represents your experience for each statement. | - Yes - No - [If yes], was it within the past 30 days?   - Yes   - No | Sexual Minority Adolescent Stress Inventory (SMASI) (64-item version) [3] | Changed “Yes” response to stand-alone responses of “Yes, within 30 days” and “Yes, not within 30 days.” |
| Minority Stress | Anticipated Discrimination | 43. These questions are about experiences related to who you are. This includes both how you describe yourself and how others might describe you. For example, your skin color, ancestry, nationality, religion, gender, sexuality, age, weight, disability or mental health issue, and income. | - Strongly agree - Agree - Neither agree nor disagree - Disagree - Strongly disagree | Intersectional Discrimination Index (Anticipated) [4] | No adaptations |
|  |  |  |  |  |  |
| Social Spaces and Cues | House smoking rules | 44. Which statement best describes the rules about smoking INSIDE YOUR HOME? | - No one is allowed to smoke anywhere INSIDE YOUR HOME - Smoking is allowed in some places or at some times INSIDE YOUR HOME - Smoking is permitted anywhere INSIDE YOUR HOME | The Tobacco Use Supplement to the Current Population Survey (TUS-CPS) [5] | Added an “other rules” write-in response option |
| Social Spaces and Cues | House vaping rules | 45. Which statement best describes the rules about vaping or using e-cigarettes INSIDE YOUR HOME? | - No one is allowed to vape anywhere INSIDE YOUR HOME - Vaping is allowed in some places or at some times INSIDE YOUR HOME - Vaping is permitted anywhere INSIDE YOUR HOME | The Tobacco Use Supplement to the Current Population Survey (TUS-CPS) [5] | Added an “other rules” write-in response option |
| Social Spaces and Cues | Household product ownership (cigarettes) | 46. Not including yourself, does anyone living in your home own an e-cigarette or other electronic nicotine  product? | - Yes - No | Population Assessment of Tobacco and Health (PATH) Study, Wave 7, Youth / Parent Questionnaire Data [1] | Adapted for combustible cigarettes |
| Social Spaces and Cues | Household product ownership (e-cigarettes) | 47. Not including yourself, does anyone living in your home own an e-cigarette or other electronic nicotine  product? | - Yes - No | Population Assessment of Tobacco and Health (PATH) Study, Wave 7, Youth / Parent Questionnaire Data [1] | No adaptations |
| Social Spaces and Cues | Conversation starters (cigarettes) | 48. Bumming a cigarette makes it easier for me to start a conversation with someone I don't know very well. | - Yes - No | Johnson et al., 2005 [6] | Changed “bumming” to “asking someone for a combustible cigarette” |
| Social Spaces and Cues | Conversation starters (e-cigarettes) | 49. Bumming a cigarette makes it easier for me to start a conversation with someone I don't know very well. | - Yes - No | Johnson et al., 2005 [6] | Changed “bumming” to “asking someone for a combustible cigarette” and asked about e-cigarettes instead |
| Social Spaces and Cues | Public comfort (cigarette use) | 50. How comfortable are you smoking combustible cigarettes in public? | - Very comfortable - Comfortable - Neither comfortable nor uncomfortable - Uncomfortable - Very uncomfortable - I don’t know - I prefer not to answer | N/A; original measure | |
| Social Spaces and Cues | Public comfort (e-cigarette use) | 51. How comfortable are you using e-cigarettes in public? | - Very comfortable - Comfortable - Neither comfortable nor uncomfortable - Uncomfortable - Very uncomfortable - I don’t know - I prefer not to answer | N/A; original measure | |
| Social Spaces and Cues | Friends’ uses (cigarettes) | 52. How many of your friends use combustible cigarettes? | - None - A few - Some - Most - All - I don’t know - I prefer not to answer | N/A; original measure | |
| Social Spaces and Cues | LGBTQIA+ friends (cigarettes) | 53. Do any of your friends that use combustible cigarettes identify as LGBTQIA+? | - No, none - Yes, a few - Yes, some - Yes, most - Yes, all - I don’t know - I prefer not to answer | N/A; original measure | |
| Social Spaces and Cues | Friends’ uses (e-cigarettes) | 54. How many of your friends use e-cigarettes? | - None - A few - Some - Most - All - I don’t know - I prefer not to answer | N/A; original measure | |
| Social Spaces and Cues | LGBTQIA+ friends (e-cigarettes) | 55. Do any of your friends who use e-cigarettes identify as LGBTQIA+? | - No, none - Yes, a few - Yes, some - Yes, most - Yes, all - I don’t know - I prefer not to answer | N/A; original measure | |
| Social Spaces and Cues | Feeling closer (cigarettes) | 56. Sharing cigarettes helps make me feel closer to other people | - None | Johnson et al., 2005 [6] | Added response options:   - Not true of me at all - Somewhat true - True - Very true of me - I don’t know - I prefer not to answer |
| Social Spaces and Cues | Partying (cigarettes) | 57. I need to smoke when I am partying | - None | Johnson et al., 2005 [6] | Added response options:   - Not true of me at all - Somewhat true - True - Very true of me - I don’t know - I prefer not to answer |
| Social Spaces and Cues | Socializing (cigarettes) | 58. I use combustible cigarette products more when I am with other people | - None | Johnson et al., 2005[6] | Added response options:   - Not true of me at all - Somewhat true - True - Very true of me - I don’t know - I prefer not to answer |
| Social Spaces and Cues | Feeling closer (e-cigarettes) | 59. Sharing e-cigarettes helps make me feel closer to other people | - None | Johnson et al., 2005 [6] | Added response options:   - Not true of me at all - Somewhat true - True - Very true of me - I don’t know - I prefer not to answer |
| Social Spaces and Cues | Partying (e-cigarettes) | 60. I need to vape when I am partying | - None | Johnson et al., 2005 [6] | Added response options:   - Not true of me at all - Somewhat true - True - Very true of me - I don’t know - I prefer not to answer |
| Social Spaces and Cues | Socializing (e-cigarettes) | 61. I use e-cigarette products more when I am with other people | - None | Johnson et al., 2005 [6] | Added response options:   - Not true of me at all - Somewhat true - True - Very true of me - I don’t know - I prefer not to answer |
|  |  |  |  |  |  |
| Purchasing | Cigarette purchasing | 62. Where do [they/you] buy your cigarettes most of the time? | - A convenience store or gas station - A supermarket, grocery store, or drug store - A warehouse club, such as Sam's or Costco - A smoke shop, tobacco specialty store, or tobacco outlet store - A duty-free shop or military commissary - A bar, pub, restaurant, or casino - A friend or relative - A swap meet or flea market - A store on an Indian reservation - A liquor store - Somewhere else | Population Assessment of Tobacco and Health (PATH) Study, Wave 7, Youth / Parent Questionnaire [1] | Changed the pronoun to just “You” and expanded response options:   - A vape shop or vapor lounge - A smoke shop, tobacco specialty store, or tobacco outlet store - A liquor store - A mall kiosk - A convenience store or gas station - A supermarket, grocery store, or pharmacy - A warehouse club, such as Sam’s or Costco - A duty-free shop or military commissary - A friend or relative - A swap meet or flea market - Online - Somewhere else (please specify) - I don’t but my combustible cigarettes - I don’t know - I prefer not to answer |
| Purchasing | E-cigarette purchasing | 63. Where do [they/you] buy your [EPRODFILL2] most of the time | - A mall kiosk - A convenience store or gas station - A supermarket, grocery store, or drug store - A warehouse club, such as Sam's or Costco - A smoke shop, tobacco specialty store, or tobacco outlet store - A duty-free shop or military commissary - A bar, pub, restaurant, or casino - A friend or relative - A swap meet or flea market | Population Assessment of Tobacco and Health (PATH) Study, Wave 7, Youth / Parent Questionnaire [1] | Changed the pronoun to just “You”, asked about c-cigarettes specifically, and expanded response options:   - A vape shop or vapor lounge - A smoke shop, tobacco specialty store, or tobacco outlet store - A liquor store - A mall kiosk - A convenience store or gas station - A supermarket, grocery store, or pharmacy - A warehouse club, such as Sam’s or Costco - A duty-free shop or military commissary - A friend or relative - A swap meet or flea market - Online - Somewhere else (please specify) - I don’t but my combustible cigarettes - I don’t know - I prefer not to answer |
| Purchasing | Difficulty buying cigarettes | 64. How easy do you think it is for people your age to buy tobacco products in a store (not including  e-cigarettes or other electronic nicotine products)? | - Very easy - Somewhat easy - Somewhat difficult - Very difficult | Population Assessment of Tobacco and Health (PATH) Study, Wave 7, Youth / Parent Questionnaire [1] | Asked in general rather than a store and asked about combustible cigarettes specifically; changed “somewhat” to “fairly” |
| Purchasing | Difficulty buying e-cigarettes | 65. How easy do you think it is for people your age to buy tobacco products in a store (not including  e-cigarettes or other electronic nicotine products)? | - Very easy - Somewhat easy - Somewhat difficult - Very difficult | Population Assessment of Tobacco and Health (PATH) Study, Wave 7, Youth / Parent Questionnaire [1] | Asked in general rather than a store and asked about e-cigarettes instead; changed “somewhat” to “fairly” |
| Purchasing | School (cigarettes) | 66. Can you purchase combustible cigarettes near your school? | - Yes - No - I don’t know - I prefer not to answer | N/A; original measure | |
| Purchasing | School (e-cigarettes) | 67. Can you purchase e-cigarettes near your school? | - Yes - No - I don’t know - I prefer not to answer | N/A; original measure | |
|  |  |  |  |  |  |
| Marketing | Product ads | 68. When you are using the Internet, how often do you see ads or promotions for e-cigarettes? (example) | - I do not use the internet - Never - Rarely - Sometimes - Most of the time - Always | National Youth Tobacco Survey (NYTS) 2022 Questionnaire [2] | Combined several media questions into a mark-all-that-apply question, asking about past 30 advertisement exposure to any tobacco and nicotine products, specifying if the products were combustible cigarettes or e-cigarettes. Response options included:   - I haven’t seen any advertisements in the past 30 days - At gas stations, convenience stores, or other retail stores - On billboards - On social media - On other websites online - On television - In newspapers or magazines - On radio - At events such as fairs, festivals, or sporting events - At nightclubs or concerts - Somewhere else (please specify) - I don’t know - I prefer not to answer |
| Marketing | Social media accounts | 69. Can you please list social media accounts that you follow that display any tobacco and nicotine content? Please list each account name on a separate line and list the account handle next to each name. Please include the social media platform as well. | - (Free response) | N/A; original measure | |
|  |  |  |  |  |  |
| Motivations | Reason for product use | 70. What was your main reason for initiation of smoking? | - Stimulation - Relaxation/enjoyment - Stress/tension - Habit/activity - Friends/social - Others | Khatteb et al, 2017 [7] | Asked about smoking and vaping and asked participants to specify motivators for both activities; added response options:   - I don’t smoke/vape - Relaxation or enjoyment - To cope with stress or tension - It’s a habit - Friends or social reasons - To connect with LGBTQIA+ people - Because smoking/vaping is cool - I’m trying to quit smoking/vaping - Other (please specify) - I don’t know - I prefer not to answer |
| Motivations | Product enjoyment | 71. What are some of the reasons you enjoy using combustible cigarettes and/or e-cigarettes? Please select all that apply (for both cigarettes and e-cigarettes). | - I don’t smoke/vape - They’re easy to get - They come in flavors I like - Other (please specify) - I don’t know - I prefer not to answer | N/A; original measure | |
| Motivations | Perceived benefits (cigarettes) | 72. [Perceived benefits of e-cigarette construct] | - Vaping helps me feel better if I have been feeling down - Even when I feel good, vaping helps me feel better - Vaping helps me think better | Bold et al., 2018 [8] | Replaced the second item with “I would feel alone without my combustible cigarette” and made the items into a Likert scale. Response options were:   - Not true of me at all - Somewhat true of me - Neither true nor false about me - Somewhat false about me - Not false of me at all - I don’t know - I prefer not to answer |
| Motivations | Perceived benefits (e-cigarettes) | 73. [Perceived benefits of e-cigarette construct] | - Vaping helps me feel better if I have been feeling down - Even when I feel good, vaping helps me feel better - Vaping helps me think better | Bold et al., 2018 [8] | Replaced the second item with “I would feel alone without my e-cigarette” and made the items into a Likert scale. Response options were:   - Not true of me at all - Somewhat true of me - Neither true nor false about me - Somewhat false about me - Not false of me at all - I don’t know - I prefer not to answer |
| Motivations | Feeling better (cigarettes and e-cigarettes) | 74. [Perceived benefits of e-cigarette construct] | - Vaping helps me feel better if I have been feeling down - Even when I feel good, vaping helps me feel better - Vaping helps me think better | Bold et al., 2018 [8] | Asked the second question separately, asking about both combustible cigarettes and e-cigarettes separately in a Likert scale. Prompts stated “Even when I feel good, smoking combustible cigarettes / using e-cigarettes helps me feel better” Response options included:   - Not true of me at all - Somewhat true of me - Neither true nor false about me - Somewhat false about me - Not false of me at all - I don’t know - I prefer not to answer |
| Motivations | Withdrawal symptoms (cigarettes and e-cigarettes) | 75. [Withdrawal symptoms of e-cigarette construct] | - After not vaping for a while, I need to vape to avoid any discomfort - When I have not used an e-cigarette for a while or tried to stop vaping, I feel more nervous/restless/anxious because I could not vape - When I go too long without vaping, I feel impatient or irritable | Bold et al., 2018 [8] | Turned the first item into a Likert scale, asking about combustible cigarettes (via smoking) and e-cigarettes separately. Response options included:   - I don’t smoke/vape - Never - Rarely - Sometimes - Often - Almost always - I don't know - Prefer not to answer |
|  |  |  |  |  |  |
| Dependency | Nicotine dependence | 76. Please respond to each question or statement by marking one box per row. | - Never - Rarely - Sometimes - Often - Always | Smoking: Nicotine Dependence for Daily and Nondaily Smokers  – Short Form 4a [9] | Changed “Always” to “Almost always” |
| Dependency | Nicotine addiction | 77. None | - Yes - No | Revised Hooked on Nicotine Checklist (HONC) [10] | No adaptations |
| Dependency | Quitting attempts | 78. Which products have you tried quitting, even once? Please select all that apply. | - Combustible cigarettes - E-cigarettes - Neither - Other products (please specify) - I don’t know - I prefer not to answer | N/A; original measure | |
| Dependency | Quitting attempt (cigarettes) | 79. In the past 12 months, have you stopped using all tobacco products for one day or longer because you were  trying to quit for good? | - Yes - No | Population Assessment of Tobacco and Health (PATH) Study, Wave 7, Youth / Parent Questionnaire [1] | Asked about combustible cigarettes specifically and how long ago the participant stopped using them. Response options included   - 1 day - 1 week - 1 month - 3 months or longer - I don’t know - I prefer not to answer |
| Dependency | Quitting attempt (e-cigarettes) | 80. In the past 12 months, have you stopped using all tobacco products for one day or longer because you were  trying to quit for good? | - Yes - No | Population Assessment of Tobacco and Health (PATH) Study, Wave 7, Youth / Parent Questionnaire [1] | Asked about e-cigarettes specifically and how long ago the participant stopped using them. Response options included   - 1 day - 1 week - 1 month - 3 months or longer - I don’t know - I prefer not to answer |
| Dependency | Product craving | 81. Please answer this statement on a scale from 0 (Strongly Disagree) to 100 (Strongly Agree) | - 1 (Strongly disagree) - 2 - 3 - 4 (Neither agree nor disagree) - 5 - 6 - 7 (strongly agree) | 10-item Questionnaire on Smoking Urges-Brief [11] | - Pulled the first item “I have a desire for a cigarette right now” and asked which products the participant had a desire to use. Response options included   - Combustible cigarettes   - E-cigarettes   - Cannabis (marijuana, pot, weed)   - Cigars, cigarillos, and bidis   - Hookah   - Smokeless (chews, snus, and snuff)   - Nicotine replacements (lozenges, patches, and gums)   - None   - I don’t know   - I prefer not to answer |
|  |  |  |  |  |  |
| Mood | General mood | 82. Indicate to what extent you GENERALLY feel this way, that is how you feel ON AVERAGE | - Very slightly - A little - Moderately - Quite a bit - Extremely | Positive and Negative Affect Scale (10-item version [12] | No adaptations |

Reference

1. National Addiction & HIV Data Archive Program. Population Assessment of Tobacco and Health (PATH) Study [United States] Public-Use Files (ICPSR 36498). Ann Arbor, MI: Inter-university Consortium for Political and Social Research; 2025. Accessed February 2, 2025.
2. Office on Smoking and Health. 2022 National Youth Tobacco Survey: Methodology Report. Atlanta, GA: US Dept of Health and Human Services, Centers for Disease Control and Prevention, National Center for Chronic Disease Prevention and Health Promotion; 2022.
3. Schrager SM, Goldbach JT, Mamey MR. Development of the Sexual Minority Adolescent Stress Inventory. Front Psychol. 2018;9:319. doi:10.3389/fpsyg.2018.00319
4. Scheim AI, Bauer GR. The Intersectional Discrimination Index: Development and validation of measures of self-reported enacted and anticipated discrimination for intercategorical analysis. Soc Sci Med. 2019;226:225-235. doi:10.1016/j.socscimed.2018.12.016
5. US Dept of Commerce, Census Bureau. National Cancer Institute and Food and Drug Administration Co-sponsored Tobacco Use Supplement to the Current Population Survey, 2018–2019. Washington, DC: US Dept of Commerce; 2020. Accessed June 18, 2025. https://cancercontrol.cancer.gov/tus-cps
6. Johnson JL, Ratner PA, Tucker RS, et al. Development of a multidimensional measure of tobacco dependence in adolescence. Addict Behav. 2005;30(3):501-515. doi:10.1016/j.addbeh.2004.07.006
7. Khattab AM, AbdelFattah EB, Abozahra AKEA. Study of smoking habit among soldiers in Cairo Security Forces Hospital. Egypt J Chest Dis Tuberc. 2017;66(2):267-277. doi:10.1016/j.ejcdt.2016.10.012
8. Bold KW, Sussman S, O'Malley SS, et al. Measuring E-cigarette dependence: Initial guidance. Addict Behav. 2018;79:213-218. doi:10.1016/j.addbeh.2017.11.015
9. Tucker JS, Shadel WG, Edelen MO, et al. Development of the PROMIS Social Motivations for Smoking item banks. Nicotine Tob Res. 2014;16(Suppl 3):S241-S249. doi:10.1093/ntr/ntt283
10. DiFranza JR, Savageau JA, Fletcher K, et al. Measuring the loss of autonomy over nicotine use in adolescents. Arch Pediatr Adolesc Med. 2002;156(4):397-403. doi:10.1001/archpedi.156.4.397
11. Cox LS, Tiffany ST, Christen AG. Evaluation of the brief questionnaire of smoking urges (QSU-brief) in laboratory and clinical settings. Nicotine Tob Res. 2001;3(1):7–16. doi:10.1080/14622200124218
12. Thompson ER. Development and validation of an internationally reliable short-form of the positive and negative affect schedule (PANAS). J Cross Cult Psychol. 2007;38(2):227-242. doi:10.1177/0022022106297301
