# Supplementary material for: Measuring Stress, Socialization, and Smoking Behaviors Among Lesbian, Gay, Bisexual, Transgender, Queer, and Other Sexual and Gender Minority Adolescents (the Puff Break Research Study): Protocol for a Ecological Momentary Assessment Study
Source: JMIR Res Protoc. 2025 Jul 3;14:e71927. doi: 10.2196/71927 (PMC12271964; doi:10.2196/71927)
Supplement: Multimedia Appendix 2 [file resprot_v14i1e71927_app2.docx]

**Appendix 2: EMA Survey Measures and Adaptations (When Applicable) in the Puff Break Study**

| **Domain** | **Measures** | **Question/Prompt/Construct** | **Response Options/Items** | **Source** | **Adaptations** |
| --- | --- | --- | --- | --- | --- |
| Present Mood | Mood | 2. What is your overall feeling right now? | - 1 (very unpleasant) - 2 - 3 - 4 - 5 - 6 - 7 - 8 - 9 (very pleasant) | Circumplex Model of Affect [1-2] | No adaptations |
| Present Mood | Mood | 3. What is your overall energy level right now? | - 1 (very low) - 2 - 3 - 4 - 5 - 6 - 7 - 8 - 9 (very high) | Circumplex Model of Affect [1-2] | No adaptations |
|  |  |  |  |  |  |
| Present Stress | Stress | 4. How stressed are you feeling right now | - 1 (not at all) - 2 - 3 - 4 (very stressed) | Do et al, 2021 [3]  Ebesutani et al, 2012 [4]  Osika et al, 2007 [5]  Cohen et al, 1983 [6] | No adaptations |
| Present Stress | Stress | 5. Please answer the prompt: I can manage with all of the things I have to do right now. | - Yes - No | Do et al, 2021 [3]  Ebesutani et al, 2012 [4]  Osika et al, 2007 [5]  Cohen et al, 1983 [6] | No adaptations |
| Present Stress | Stress | 6. Please answer the prompt: I can manage with all of the things I have to do right now. | - Yes - No | Do et al, 2021 [3]  Ebesutani et al, 2012 [4]  Osika et al, 2007 [5]  Cohen et al, 1983 [6] | No adaptations |
|  |  |  |  |  |  |
| Present Safety | Safety | 7. How safe or unsafe do you feel right now? Please select one. | - Very safe - Safe - Kind of safe - Neither safe nor unsafe - Kind of unsafe - Unsafe - Very unsafe | N/A; original measure | |
|  |  |  |  |  |  |
| Present Support | Stress | 8. How supported or unsupported do you feel right now? Please select one. | - Very supported - Supported - Kind of supported - Neither supported nor unsupported - Kind of unsupported - Unsupported - Very unsupported | N/A; original measure | |
|  |  |  |  |  |  |
| Present Craving | Product craving | 9. Please answer this statement on a scale from 0 (Strongly Disagree) to 100 (Strongly Agree) | - 1 (Strongly disagree) - 2 - 3 - 4 (Neither agree nor disagree) - 5 - 6 - 7 (strongly agree) | 10-item Questionnaire on Smoking Urges-Brief [7] | Pulled the first item “I have a desire for a cigarette right now” and asked which products the participant had a desire to use. Response options included   - Combustible products - Smokeless products - Vaporized products - Cannabis products - Other products (please specify) - None of these products - I don’t know - I prefer not to answer |
|  |  |  |  |  |  |
| Present Tobacco and Nicotine Use | Product use (current) | 10. You mentioned that you started using [LIST OF ALL PRODUCTS INITIATED/USED AT EARLIEST AGE]  [in the past 12 months/at age {EARLIEST AGE}]. Which type of tobacco did you try first? | - Cigarettes - E-cigarettes or other electronic nicotine products - Traditional cigars - Cigarillos - Filtered cigars - Shisha or hookah tobacco - Snus - Smokeless tobacco (such as moist snuff, dip, spit, or chew) - IQOS - Other (specify) - Don’t know - Refused | Population Assessment of Tobacco and Health (PATH) Study, Wave 7, Youth / Parent Questionnaire [8] | Asked about product use in the moment and condensed repose options:   - - Combustible products   - Smokeless products   - Vaporized products   - Cannabis products   - Other products (please specify)   - None of these products   - I don’t know   - I prefer not to answer |
|  |  |  |  |  |  |
| Present Other Substance Use | Alcohol use (current) | 11. In the past 12 months, have you used any alcohol, including small sips or tastes? | - Yes - No | Population Assessment of Tobacco and Health (PATH) Study, Wave 7, Youth / Parent Questionnaire [8] | Asked about alcohol use in the moment. |
| Present Other Substance Use | Drug use (current) | 12. In the past 12 months, have you used any of the following prescription drugs that were not  prescribed for you or that you took only for the experience or feeling they caused? | - Yes - No | Population Assessment of Tobacco and Health (PATH) Study, Wave 7, Youth / Parent Questionnaire [8] | Asked about other substances in general and about other substances used at the moment. |
| Present Other Substance Use | Other substances | 13. *[If “Yes” to Q12]* What other substance(s) are you using right now? Please list them. | - (Free response) | N/A; original measure | |
|  |  |  |  |  |  |
| Present Socialization | Place (current) | 14. Where are you right now? | - Home - Work - Vehicle - Other’s home - Bar - Restaurant - Other place | Nguyen et al., 2018 [9] | Removed “bar” and added response options from Aim 1 findings:   - Home - Dorm - School - Work - Vehicle - Other’s home - Park - Outside (not park) - Restaurant - Bus stop - Party - Event - LGBTQ+ designated space - Other place - I don’t know - I prefer not to answer |
| Present Socialization | Product use rules (current) | 15. Is smoking banned at your current location? | - Yes - No | Nguyen et al., 2018 [9] | The question asked if tobacco and nicotine products were allowed to be used in the places where the participant currently was, and responses were changed from a “yes/no” question to a Likert scale:   - Yes, everywhere - Yes, almost everywhere - Sometimes, depends on the place - No, almost nowhere - No, nowhere - I don’t know - I prefer not to answer |
| Present Socialization | People with | 16. Who are you currently with? | - Unknown persons - Family members - Friends - Acquaintances - Coworkers - Partner | Nguyen et al., 2018 [9] | Added “other” |
| Present Socialization | Type of people with | 17. *If “Yes” to “Other” on Q16]* What other persons are you with right now? | - Free response | N/A; original measure | |
| Present Socialization | LGBTQ persons | 18. How many of these people that you are with right now identify as LGBTQIA+? Please select one | - None - Not many - Some - Most - All - I don’t know - I prefer not to answer | N/A; original measure | |
| Present Socialization | Nguyen et al., 2018 | 19. Is there a presence of other smokers? | - Yes - No | No adaptations | |
| Present Socialization | Other persons’ product use | 20. You mentioned that you started using [LIST OF ALL PRODUCTS INITIATED/USED AT EARLIEST AGE]  [in the past 12 months/at age {EARLIEST AGE}]. Which type of tobacco did you try first? | - Cigarettes - E-cigarettes or other electronic nicotine products - Traditional cigars - Cigarillos - Filtered cigars - Shisha or hookah tobacco - Snus - Smokeless tobacco (such as moist snuff, dip, spit, or chew) - IQOS - Other (specify) - Don’t know - Refused | Population Assessment of Tobacco and Health (PATH) Study, Wave 7, Youth / Parent Questionnaire [8] | Asked the participant what products others were currently using around them; condense response options:   - Combustible products - Smokeless products - Vaporized products - Cannabis products - Other products (please specify) - None of these products - I don’t know - I prefer not to answer |
|  |  |  |  |  |  |
| Past Minority Stress | Minority stress | 21. In your day-to-day life, how often do any of the following things happen to you? | In your day-to-day life, how often do any of the following things happen to you?  1. You are treated with less courtesy than other people are.  2. You are treated with less respect than other people are.  3. You receive poorer service than other people at restaurants or stores.  4. People act as if they think you are not smart.  5. People act as if they are afraid of you.  6. People act as if they think you are dishonest.  7. People act as if they’re better than you are.  8. You are called names or insulted.  9. You are threatened or harassed. | Everyday Discrimination Scale [10] | Did not ask follow-up questions about identity; made the question a “select-all-that apply” rather than ask about the frequency of each experience.  “The following questions refer to your identity, which means any and all of your identities such as your gender, race, ethnicity, sexual orientation, social class, religion, nationality, body size, or any other identities. If any of the events listed below happened to you between Time A and the time you went to Time B, please select all that apply.”  Responses were reworded:   - I was targeted or harassed because of my identity - I saw or heard negative, hurtful, or offensive messages or stereotypes about my identity or people with the same identity - I was ignored, isolated, or made to feel invisible because of my identity - I was misunderstood because of my identity - People stared at me because of my identity - I was not accepted because of my identity - Someone made me feel uncomfortable or unsafe because of my identity - My identity interfered with my life - Someone made me feel less of a human because of my identity - None of these events happened to me - I don’t know - I prefer not to answer |
| Past Tobacco and Nicotine Use | Product use (past) | 22. You mentioned that you started using [LIST OF ALL PRODUCTS INITIATED/USED AT EARLIEST AGE]  [in the past 12 months/at age {EARLIEST AGE}]. Which type of tobacco did you try first? | - Cigarettes - E-cigarettes or other electronic nicotine products - Traditional cigars - Cigarillos - Filtered cigars - Shisha or hookah tobacco - Snus - Smokeless tobacco (such as moist snuff, dip, spit, or chew) - IQOS - Other (specify) - Don’t know - Refused | Population Assessment of Tobacco and Health (PATH) Study, Wave 7, Youth / Parent Questionnaire [8] | Asked about product use between Time A and Time B. Also asked about product use in the moment and condensed repose options:   - - Combustible products   - Smokeless products   - Vaporized products   - Cannabis products   - Other products (please specify)   - None of these products   - I don’t know   - I prefer not to answer |
| Past Tobacco and Nicotine Use | Amount of past combustible product use | 23. Between [Time A] and [Time B], how many times did you use combustible products? | - [half-hour increments between Time A and Time B] | N/A; original measure | |
| Past Tobacco and Nicotine Use | Time of last use of combustible products | 24. Between [Time A] and [Time B], how many times did you use combustible products? | - [half-hour increments between Time A and Time B] | N/A; original measure | |
| Past Tobacco and Nicotine Use | Amount of past smokeless product use | 25. Between [Time A] and [Time B], how many times did you use smokeless products? | - [half-hour increments between Time A and Time B] | N/A; original measure | |
| Past Tobacco and Nicotine Use | Time of last use of smokeless products | 26. Between [Time A and [Time B] what time did you last use smokeless products? Please select one time frame below. | - [half-hour increments between Time A and Time B] | N/A; original measure | |
| Past Tobacco and Nicotine Use | Amount of past vaporized product use | 27. Between [Time A] and [Time B], how many times did you use vaporized products? | - [half-hour increments between Time A and Time B] | N/A; original measure | |
| Past Tobacco and Nicotine Use | Time of last use of vaporized products | 28. Between [Time A] and [Time B] what time did you last use vaporized products? Please select one time frame below. | - [half-hour increments between Time A and Time B] | N/A; original measure | |
| Past Tobacco and Nicotine Use | Time of last use of cannabis products | 29. Between [Time A] and [Time B], how many times did you use cannabis products? | - [half-hour increments between Time A and Time B] | N/A; original measure | |
| Past Tobacco and Nicotine Use | Time of last use of cannabis products | 30. Between [Time A] and [Time B] what time did you last use cannabis products? Please select one time frame below. | - [half-hour increments between Time A and Time B] | N/A; original measure | |
| Past Tobacco and Nicotine Use | Time of last use of other products | 31. Between [Time A] and [Time B], how many times did you use other products? | - [half-hour increments between Time A and Time B] | N/A; original measure | |
| Past Tobacco and Nicotine Use | Time of last use of other products | 32. Between [Time A] and [Time B] what time did you last use other products? Please select one time frame below. | - [half-hour increments between Time A and Time B] | N/A; original measure | |
| Past Tobacco and Nicotine Use | E-cigarette flavor | 33. In the past 30 days, were any of the e-cigarettes you used flavored? Please select all that apply.  33. What flavors were the e-cigarettes that you have used in the past 30 days? (Select one or more) | - Yes - No - Don’t Know - Menthol - Mint - Clove or spice - Fruit - Chocolate - Alcoholic drinks (such as wine, margarita, or other cocktails) - Candy, desserts, or other sweets - Some other flavor not listed here (Specify:) | National Youth Tobacco Survey (NYTS) 2022 Questionnaire [11] | Changed to ask whether or not products used in the past (between Time A and Time B) were flavored or not.   - Yes, tobacco flavor - Yes, flavors other than tobacco - No, no flavor |
| Past Tobacco and Nicotine Use | Place (past) | 34. Where are you right now? | - Home - Work - Vehicle - Other’s home - Bar - Restaurant - Other place | Nguyen et al., 2018 [9] | Asked about where any products were used between Time A and Time B; removed “bar” and added response options from Aim 1 findings:   - Home - Dowm - School - Work - Vehicle - Other’s home - Park - Outside (not park) - Restaurant - Bus stop - Party - Event - LGBTQ+ designated space - Other place - I don’t know - I prefer not to answer |
| Past Tobacco and Nicotine Use | Product use rules (past) | 35. Is smoking banned at your current location? | - Yes - No | Nguyen et al., 2018 [9] | The question asked if tobacco and nicotine products were allowed to be used in the places where the participant currently was, and responses were changed from a “yes/no” question to a Likert scale:   - Yes, everywhere - Yes, almost everywhere - Sometimes, depends on the place - No, almost nowhere - No, nowhere - I don’t know - I prefer not to answer |
| Past Tobacco and Nicotine Use | Ofteness of using products with other people | 36. Between [Time A] and [Time B], how often did you use these tobacco and nicotine products with other people? Please select one. | - I was always alone - I was rarely with other people - I was with other people sometimes - I was often with other people - I was almost always with other people - I don’t know - I prefer not to answer | N/A; original measure | |
| Past Tobacco and Nicotine Use | How many of the other persons identify as LGBTQIA+ | 37. Between [Time A] and [Time B], how many of these people that you used tobacco and nicotine products with also identify as LGBTQIA? Please select one. | - None - Not many - Some - Most - All - I don’t know - I prefer not to answer | N/A; original measure | |
| Past Tobacco and Nicotine Use | The products others were using | 38. Between [Time A] and [Time B] what tobacco and nicotine products were others using? Please select all that apply. | - Combustible products - Smokeless products - Vaporized products - Cannabis products - Other products (please specify) - None of these products - I don’t know - I prefer not to answer | N/A; original measure | |
| Past Other Substances | Alcohol use (past) | 39. In the past 12 months, have you used any alcohol, including small sips or tastes? | - Yes - No | Population Assessment of Tobacco and Health (PATH) Study, Wave 7, Youth / Parent Questionnaire [8] | Asked about alcohol use between Time A and Time B |
| Past Other Substances | Drug use (past) | 40. In the past 12 months, have you used any of the following prescription drugs that were not  prescribed for you or that you took only for the experience or feeling they caused? | - Yes - No | Population Assessment of Tobacco and Health (PATH) Study, Wave 7, Youth / Parent Questionnaire [8] | Asked about other substances in general and asked about other substance use between Time A and Time B |
| Past Other Substances | Other substances | 41. *[If “Yes” to Q40]* What other substance(s) are you using right now? Please list them. | - (Free response) | N/A; original measure | |
| Past Media | Media exposure | 42. Between [Time A] and [Time B], have you seen any media encouraging or discouraging tobacco, nicotine, or cannabis product use, virtually or in person? | - Yes, encouraging - Yes, discouraging - No, I have not seen any | N/A; original measure | |
| Past Media | Encouraging media | 43. *[If “Yes, encouraging” to Q41]* Between [Time A] and [Time B], have you seen any media encouraging or discouraging tobacco, nicotine, or cannabis product use, virtually or in person? | - Digital ads or commercials - Social media posts - Videos or images - Fliers - Billboards - None - I prefer not to answer | N/A; original measure | |
| Past Media | Discouraging media | 44. *[If “Yes, discouraging” to Q41]* Between [Time A] and [Time B], have you seen any media encouraging or discouraging tobacco, nicotine, or cannabis product use, virtually or in person? | - Digital ads or commercials - Social media posts - Videos or images - Fliers - Billboards - None - I prefer not to answer | N/A; original measure | |

References

1. Posner J, Russell JA, Peterson BS. The circumplex model of affect: an integrative approach to affective neuroscience, cognitive development, and psychopathology. Dev Psychopathol. 2005;17(3):715-734. doi:10.1017/S0954579405050340
2. Sharar SR, Alamdari A, Hoffer C, Hoffman HG, Jensen MP, Patterson DR. Circumplex model of affect: a measure of pleasure and arousal during virtual reality distraction analgesia. Games Health J. 2016;5(3):197-202. doi:10.1089/g4h.2015.0046
3. Do B, Mason TB, Yi L, Yang CH, Dunton GF. Momentary associations between stress and physical activity among children using ecological momentary assessment. Psychol Sport Exerc. 2021;55:101935. doi:10.1016/j.psychsport.2021.101935
4. Ebesutani C, Regan J, Smith A, Reise S, Higa-McMillan C, Chorpita BF. The 10-item positive and negative affect schedule for children, child and parent shortened versions: application of item response theory for more efficient assessment. J Psychopathol Behav Assess. 2012;34(2):191–203. doi:10.1007/s10862-011-9273-2
5. Osika W, Friberg P, Wahrborg P. A new short self-rating questionnaire to assess stress in children. Int J Behav Med. 2007;14(2):108–117. doi:10.1007/BF03004176
6. Cohen S, Kamarck T, Mermelstein R. A global measure of perceived stress. J Health Soc Behav. 1983;24(4):385-396. doi:10.2307/2136404
7. Cox LS, Tiffany ST, Christen AG. Evaluation of the brief questionnaire of smoking urges (QSU-brief) in laboratory and clinical settings. Nicotine Tob Res. 2001;3(1):7–16. doi:10.1080/14622200124218
8. National Addiction & HIV Data Archive Program. Population Assessment of Tobacco and Health (PATH) Study [United States] Public-Use Files (ICPSR 36498). Ann Arbor, MI: Inter-university Consortium for Political and Social Research; 2025. Accessed February 2, 2025.
9. Nguyen N, McQuoid J, Ramo D, Holmes LM, Ling PM, Thrul J. Real-time predictors of smoking among sexual minority and heterosexual young adults: An ecological momentary assessment study. Drug Alcohol Depend. 2018;192:51-58. doi:10.1016/j.drugalcdep.2018.07.021
10. Williams D, Yu Y, Jackson J, Anderson N. Racial differences in physical and mental health: socioeconomic status, stress, and discrimination. J Health Psychol. 1997;2(3):335-351. doi:10.1177/135910539700200305
11. Office on Smoking and Health. 2022 National Youth Tobacco Survey: Methodology Report. Atlanta, GA: US Dept of Health and Human Services, Centers for Disease Control and Prevention, National Center for Chronic Disease Prevention and Health Promotion; 2022.
